# Whole-body distribution of three *Pseudomonas* phages characterized by a translational physiologically based pharmacokinetic model

**DOI:** 10.1128/aac.01506-25

**Published:** 2025-12-05

**Authors:** Arne Echterhof, Tejas Dharmaraj, Patrick Blankenberg, Bobby Targ, Thomas D. Nguyen, Paul L. Bollyky, Nicholas M. Smith, Francis G. Blankenberg

**Affiliations:** 1 Division of Infectious Diseases and Geographic Medicine, Department of Medicine, Stanford University School of Medicine 10624 , Stanford, California, USA; 2 Institute of Medical Microbiology, University Hospital of Muenster 235555, Muenster, Germany; 3 Division of Clinical and Translational Therapeutics, School of Pharmacy & Pharmaceutical Sciences, University at Buffalo 15497, Buffalo, New York, USA; 4 Division of Pediatric Radiology and Nuclear Medicine, Department of Radiology, Lucile Packard Children’s Hospital, Stanford, California, USA; Columbia University Irving Medical Center, New York, New York, USA

**Keywords:** pharmacokinetics, physiologically based pharmacokinetic, bacteriophage, *Pseudomonas*, translational

## Abstract

Bacteriophage (phage) therapy holds great promise for treating antimicrobial-resistant infections. However, the pharmacokinetics (PK) of phage have been difficult to characterize due to a lack of standardized protocols for phage purification, labeling, and *in vivo* quantification. Here, we present robust methods for ultrapure phage preparation, as well as non-destructive, highly stable attachment of radio-iodide to phage using a well-described Sulfo-SHPP linker. We purified and radiolabeled the phage strains, PAML-31-1, OMKO1, and Luz24, lytic to drug-resistant *Pseudomonas aeruginosa,* for biodistribution assay in normal young adult CD-1 mice injected via intravenous injection. Groups of five mice were euthanized, and tissues/organs were removed for weighing and scintillation well counting of ^125^I activity. A physiologically based PK model was then constructed, focusing on compartments describing blood, lung, muscle, bone, liver, stomach, spleen, small intestines, large intestines, and kidney. Tissue partition coefficients (K_P_) were estimated for high-perfusion organs (lung and kidney) as 0.000138, GI organs (liver, spleen, and stomach) as 0.627, and all other organs as 0.220. Monte Carlo simulations predicted rapid elimination of phage in humans, with blood concentrations being <10^2^ PFU/mL by 12 h, whereas simulated multi-dose regimens and continuous infusion regimens were predicted to have sustained concentrations. Our physiologically based PK model of phage represents the first rigorous preclinical assessment of phage PK utilizing contemporary pharmacometric approaches amenable to both preclinical and clinical study design.

## INTRODUCTION

In the USA, 2.8 million individuals are infected by drug-resistant infections every year, and this number is expected to grow to over 10 million individuals per year globally by 2050 (1). Antibacterial resistance is an ongoing public health concern and has been highlighted by the United States Centers for Disease Control and Prevention (2) and the World Health Organization (3). In the nosocomial setting, multi- and extensively drug-resistant (MDR/XDR) *Pseudomonas aeruginosa* is particularly problematic due to a higher degree of antimicrobial resistance (4–6), and the all-cause mortality rates for XDR *P. aeruginosa* have exceeded 8%–30% (6–8). Unfortunately, antibacterial resistance has been exacerbated by stagnant development of mechanistically novel classes of antibiotics.

Bacteriophages—viruses that specifically attack bacteria—offer a promising solution to the antibiotic resistance crisis. Lytic phages are the primary focus for pharmaceutical development, as they rapidly lyse and kill their bacterial targets. Lytic phages were first suggested as possible antimicrobials in the early 20th century (9), but they were disfavored relative to small-molecule antibiotics due to the complexities with phage production, mechanism of action, narrow host range, pharmacokinetics (PK), and the risk for resistance development (10). Outside of their use for treating resistant bacterial infections, there is also significant interest in engineering phages for drug (11, 12) or gene delivery (13), which also supports improved quantification of phage PK to identify optimal dosing practices (14).

Despite the century-long interest in phage therapy, there is limited understanding of how phages move throughout the body, which is critical to optimize phage dosing. For conventional antibiotics, exposure-response data (15) are used to identify PK/PD indices based on key endpoints: area under the concentration-time curve to minimum inhibitory concentration ratio (AUC:MIC), the maximum concentration to minimum inhibitory concentration ratio (C_max_:MIC), or the percent time the antibiotic concentration exceeds the minimum inhibitory concentration (%T > MIC). However, for phages, nonlinear PK has been reported (16), and the phage *in vitro* susceptibility metrics are unclear. Ultimately, these unique PK/PD attributes of phage necessitate an alternate development paradigm.

The mechanisms governing phage absorption, distribution, metabolism, and elimination are still being studied, and reviews of current knowledge have been published by others (10, 15, 17–21). The overarching goal of describing phage PK is to link an administered phage dose to achieve a target tissue concentration, while accounting for sources of variability. As a first step, it is critically important to quantify phage concentrations in blood and at tissue sites under uninfected conditions. In establishing this baseline PK knowledge, future studies that are confounded by phage “auto-dosing” can be better evaluated (17). Ultimately, characterizing phage PK to quantitatively link phage dose to target site concentration will provide a rational basis for selection of the best dose and administration schedule.

The objective of this project was to utilize ^125^I-phage to assess the whole-body distribution of phage particles and to characterize the pharmacokinetics using a first-in-class physiologically based PK (PBPK) model. As a secondary objective, we sought to identify any effects of phage size on phage tissue distribution, retention, and elimination. We hypothesized that PBPK modeling can characterize generalizable features of phage PK and improve predictions of phage PK in humans. To this end, we used three different clinically relevant phages, Luz24, PAML-31-1, and OMKO1, with diverse sizes of 63 nm, 227 nm, and 400 nm, respectively. These phages have well-characterized physical (22) properties and exhibit lytic behavior against clinically isolated strains of antibiotic-resistant *Pseudomonas aeruginosa* (6, 23). Finally, we attempted to scale the model for prediction of phage dosing and target site penetration in humans to hypothesize clinical dosing strategies.

Previous work has shown that nano-scale objects’ distribution to peripheral tissues is partially dictated by their size, which ranges from 30 to >600 nm (24). However, other biophysical and immunological characteristics are also highly relevant to phage PK (25). However, developing a model for phage PK can likely utilize some of the same principles governing the nanoparticles and liposomal disposition, with additional model features to describe phage-specific biological processes.

Phage distribution is permeability-limited and dependent on tissue structure and cell-mediated active transport processes (26, 27). Phage elimination is presently considered to occur through the mononuclear phagocyte system (MPS) primarily, which is phagocyte-mediated and largely occurs in the liver and spleen (28). However, phage bound to bacterial debris (e.g., endotoxin) can trigger additional immune-mediated clearance, though the exact kinetics of these processes are still being studied (29).

Combined, the permeability-limited distribution to tissues and cellular-based elimination pathways, which are evolutionarily conserved processes, mean that phage PK could be scalable between species using physiological modeling approaches. We hypothesize that a physiologically based PK modeling approach is ideally suited to describe phage PK for preclinical assessment and scaling for first-in-human dose selection.

To date, phage whole-body distribution *in vivo* has been characterized using limited numbers of tissues (blood, liver, and spleen), sparse time points, and using data-driven non-compartmental or compartmental analytical methods, which has challenged translation to the clinic. Implementation of a PBPK model utilizing known anatomy of key organs (e.g., lungs, kidney, stomach, small bowel, large bowel, muscle, etc.) can improve phage translation. Additionally, quantifying the influence of phage size on disposition would be a new tool in the field’s search for improved understanding of phage PK, improving study designs for future preclinical, clinical, or ongoing compassionate use cases.

Constructing a PBPK model capable of achieving these goals requires quantification methods that are amenable to rich sampling schedules that can ideally quantify phage in both tissue and blood. Radiolabeling methods, such as with ^125^I, have a long tradition in pharmacology studies and have been used extensively in describing the preclinical PK of complex biologics and biotic agents. Use of ^125^I labeling of drugs for preclinical PK studies is well suited to provide detailed quantification of phage in blood and organs. Furthermore, as compared to plaque assays, radiolabeling allows for high frequency sampling of phage concentration over clinically relevant time courses for improved temporal resolution.

## MATERIALS AND METHODS

### Phage lysate purification

For these studies, we used a CIMmultus monolithic OH-column chromatography-based approach, as recently described (30, 31). In brief, the ClMmultus OH 1 mL Monolithic Hydroxyl column with 2 µm channel size (Sartorius BIA Separations, Ajdovščina, Slovenia) was installed as per manufacturer instructions to ÄKTA Pure FPLC (GE Healthcare Biosciences, Sweden) chromatography system. Clean-in-place protocols (CIPs) for the FPLC system and for the column were performed with 1 M NaOH according to the column manufacturer’s instructions. Concentrated buffer of 200 mM Tris at pH 7.5 was used to restore pH for both CIPs.

Phage lysates were treated with 5 U/mL Benzonase nuclease (Sigma Aldrich) overnight at 37°C to digest free DNA and passed through a 0.22 µm PES membrane. The column was equilibrated with 10 column volumes (CV) of 1.5 KH_2_PO_4_, pH 7.0. The phage lysate was diluted at a 1:1 ratio with 3.0 M KH_2_PO_4_ buffer balanced to pH 7.0 to match the column binding condition and loaded via the sample pump into the FPLC system. A 0%–100% linear gradient was performed over 20 CV, starting with 1.5 M KH_2_PO_4_ at pH 7.0 and ending with 20 mM KH_2_PO_4_ at pH 7.0. One milliliter fractionations of column output were analyzed for a peak in UV absorbance at 280 nm using FPLC integrated wavelength UV-vis detector (GE Healthcare Biosciences, Sweden), and fractionations at the top of the UV peak were pooled and passed through 0.22 µm PES membrane. Filtered product was considered purified phage. The column was then re-equilibrated with 10 CV of 1.5 M KH_2_PO_4_ before repeating system CIP and column CIPs as per manufacturer instructions.

The PAML-31-1 and OMKO1 lytic phages were prepared with the ÄKTA/Monolithic Hydroxyl column in a volume of ~20 mL of 1 to 5 × 10^11^ PFU/mL with LPS <0.05 and <0.5 EU/mL, respectively, measured using a recombinant Factor C Endotoxin Detection assay (bioMérieux, France, Endonext EndoZyme II assay) with an assay range from 0.005 to 50 EU/mL. The Luz24 phage lysate was unable to be loaded and purified with the ÄKTA/Monolithic Hydroxyl column under the conditions for PAML-31-1 and OMKO1 as described above. Instead, Luz24 phage lysate was purified with sequential spin/wash cycles ×10 after overnight DNase treatment of 0.22 µm filtered lysate, spiked with rh-Annexin V (NASI, 2004), 0.1 mg/mL + 2 mM CaCl_2_ (final concentrations) in HEPES buffer and 15 mL 100 kDa Amicon centrifugal filters spun 10 min at 5,000 × *g* per cycle. Two additional cycles were performed with HEPES buffer to remove Annexin V/Ca^2+^ from the phage solution. The LPS level of the purified of Luz24 stock solution was 1.89 EU/mL.

All phage samples demonstrated >98.5% reduction of proteins as measured by a bicinchoninic acid (BCA) Pierce BCA Protein Assay kit (Thermo Scientific, USA). While column purification increased DNA content post-purification in most samples, the elution of labeled phage on a NAP-5 column results in removal of >97% of nucleotides and oligonucleotides from phage samples. Note there was <30 ng/mL of DNA before radiolabeling/NAP-5 filtration as measured by a Quant-iT PicoGreen assay (Invitrogen, USA) for all three phages.

### Radio-iodination of Sulfo-SHPP and conjugation of ^125^I-Sulfo-SHPP to phage

Briefly, 15 µL (0.84 to 1.15 mCi) of stock ^125^I (0.84 to 1.15 mCi, NEN Radiochemicals) in 0.1 M NaOH (pH 12–14, reductant-free) was added to 75 µL of buffer (0.1 M Na_2_HPO_4_ + 100 mM NaCl, pH 7.2) in a 1 mL 6 × 50 mm borosilicate glass tube. Next, three doubly buffer-washed Pierce Iodination Beads were added immediately, followed by 10 µL of a freshly prepared solution of 2 mg–3 mg Sulfo-SHPP (Thermo Scientific Pierce Sulfo-SHPP, sulfosuccinimidyl-3-(4-hydroxyphenyl) propionate dissolved in 10 mL of Na_2_HPO_4_ buffer. The Sulfo-SHPP/^125^I/Iodobead mixture was then reacted for 30 min at RT.

After 30 min, the Sulfo-SHPP/^125^I mixture was aspirated and placed into a second fresh glass tube. The three Iodobeads remaining in the first glass tube were then washed twice with 50 µL of HEPES conjugation buffer (20 mM HEPES, 100 mM NaCl, pH 7.4), and the wash was placed into the second test tube containing the Sulfo-SHPP/^125^I mixture and was allowed to rest for 30 min at RT to ensure completion of the oxidation/radio-iodination reaction.

For phage conjugation, the Sulfo-SHPP/^125^I mixture (~170 to 180 µL) was added to a 0.5 mL Eppendorf tube containing 4 mL of 1 to 5 × 10^11^ PFU/mL phage suspended in HEPES buffer concentrated down to a 100 to 200 µL final volume with 100 kDa Amicon microcentrifugal filters. The conjugation reaction was allowed to proceed for 10 min with frequent gentle mixing using a pipette. After 10 min, the conjugation reaction mixture was placed into the reservoir of a NAP-5 desalting gel column (Cytiva, Amersham) equilibrated with 1× PBS buffer (pH 7.2) and allowed to flow through. An additional volume of 1× PBS was stacked on top of the conjugation reaction mixture to equal a total flow-through volume of 500 µL. Eluted solution (500 µL) was then discarded and 500 µL of 1× PBS (Fraction #1) was placed into the NAP-5 reservoir and collected, followed by 250 µL of 1× PBS (Fraction #2), which was also collected. Fractions #1 and #2 were pooled with an activity of 12.2 to 17.9 µCi of conjugated SHPP-^125^I-phage in a final volume of 2.5 mL of 1× PBS.

### *In vivo* studies

Experiments utilized healthy, 5- to 7-week-old male Swiss-Webster CD-1 mice weighing 22 to 30 g (Charles River), and stuides were performed 1 week after acclimation to housing at the VSC facility at Stanford. Housing conditions for mice consisted of a 12:12 h dark-light cycle and temperature of 22 ± 2°C with food and water *ad libitum*.

Each dose composed of 50 µL of phage stock of 2.5 mL was bolus-injected into the penile vein of mice anesthetized with IP injection of 0.1 mL of a 7:3:1 cocktail of sterile 1× PBS, ketamine (100 mg/mL), and xylazine (20 mg/mL), respectively. Mice were divided into six groups of five mice each euthanized via CO_2_ inhalation after sedation with the 7:3:1 cocktail at 30 min, 1 h, 2 h, 4 h, 8 h, and 24 h after injection of radiolabeled OMKO1 and Luz24 phage. For the PAML-31-1 group, mice were injected 30 min, 1 h, 2 h, 4 h, 24 h, and 72 h after injection of radiolabeled phage.

Blood samples were obtained by retro-orbital bleeds with 70 µL heparinized hematocrit tubes in separate groups of sedated mice at 1 min, 5 min, 15 min, or 30 min after tracer injection.

At each time point after injection, replicate mice were euthanized, dissected, and target organs were harvested, washed in 1× PBS, and weighed in sample tubes for scintillation well counting. Samples, as well as standard tubes (1 mL of a 1% injected activity), were counted for 1 min at an energy level of 0–100 keV with a window of 0–10 keV with subtraction of background radiation. Results were expressed as %ID (injected dose) or %ID/gram of tissue.

### Pharmacokinetic analysis

Data were analyzed as %ID/g for blood and all tissues, whereas the stomach contents were modeled as %ID. A PBPK model was constructed using compartments describing blood, lung, muscle, bone, liver, stomach, spleen, small intestines, large intestines, and kidney. Additionally, organ compartments describing murine skin and brain were included for hypothesis-generating purposes given the relevance for infection models, but no data were available for estimation phage PK parameters. A carcass compartment was used for mass balance of drug from other tissues not measured. All organs were modeled using a permeability-limited distribution structure, and each tissue was described as:


(1) $$\frac{dC_{\text{i,v}}}{dt}∙V_{\text{i,v}}=Q_{i}∙\left(C_{a}\left(t\right)-C_{\text{i,v}}\left(t\right)\right)-P∙Q_{i}∙\left(C_{i,v}\left(t\right)-\frac{C_{i,int}\left(t\right)}{K_{p}}\right)$$

(2) $$\frac{dC_{i,int}}{dt}\cdot V_{i,int}=P\cdot Q_{i}\cdot \left(C_{i,v}\left(t\right)-\frac{C_{i,int}\left(t\right)}{K_{p}}\right)-k_{i,up}\left(t\right)\cdot C_{i,int}\left(t\right)\cdot V_{i,int}+k_{rel}\cdot A_{\text{i,MPS}}\left(t\right)$$

Where, for tissue *i,* C_v_ is the phage concentration of the vascular space of the tissue, *C*_int_ is the phage concentration in the interstitial space of tissue, *V*_*v*_ is the vascular volume of the tissue, *V*_int_ is the volume of the interstitial space of the tissue, *P* is the permeability-surface coefficient, *Q*_*i*_ is the blood flow rate of the tissue, *k*_up_ is a first-order rate constant governing phage uptake into the MPS compartment, *k*_rel_ is a first-order rate constant governing phage release from the MPS compartment, and *A*_MPS_ is the amount of phage in the MPS compartment of the tissue.

MPS uptake was modeled as a saturable process that was dependent on the total number of tissue-resident phagocytes, maximum capacity (parameterized as %ID per 10^5^ phagocytes), and the amount of phage in the MPS compartment. This was described by the following equations:


(3) $$\frac{dA_{i,res}}{dt}=k_{i,up}(t)∙C_{i,int}(t)∙V_{i,int}-k_{rel}∙A_{\text{i,MPS}}(t)-k_{\text{deg}}∙A_{\text{i,MPS}}(t)$$

(4) $$k_{i,up}\left(t\right)=k_{\text{up,max}}∙\left(1-\frac{A_{\text{i,MPS}}(t)}{A_{\text{i,max}}}\right)$$

(5) $$A_{\text{i,max}}=M_{i}∙V_{i}∙\left(\frac{A_{{\text{MPS}}^{*}}}{10^{5}}\right)$$

(6) $$A_{MPS^{*}}=\frac{A_{MPS}}{Dose}$$

Where *A*_i,max_ is the total tissue capacity of the phage dose (%ID), *M*_*i*_ is the number of tissue-resident phagocytes per gram of tissue (phagocytes/gram), *V*_*i*_ is the total tissue volume (g), and *A*_max_ is the fixed parameter of MPS carrying capacity (%ID/10^5^ phagocytes) (26). Previously published data have shown biologically plausible transcytosis of phage across epithelial membranes, which supports that a portion of phage elimination may occur through cell-mediated processes located at the gastrointestinal surface, hepatobiliary interface, and in the kidney (26, 27).

Lung and kidney compartments were described using a unique partition coefficient due to each organ’s large blood flow rates. Equations describing this are provided in the supplemental materials. Model parameters and sources are outlined in Table S1. Briefly, mouse organ weights were fixed as fractions of total weight. Mouse organ flow rates were fixed to values based on the fraction of cardiac output, which was scaled based on mouse weight.

The model was simultaneously fit to all data from each tissue, estimating a whole-body permeability coefficient (*P*), tissue partition coefficients (*K*_*p*_), phage degradation rate in MPS compartment (*T*_MPS,deg_), and hypothetical active clearance at surface sites (*CL*_Active_). Gastric and urine transit times were empirically estimated due to experimental limitations on urine and stomach contents sample collection.

A twin PBPK model was constructed to describe *free* ^125^I produced through either thermal deconjugation of phage (a relatively slow process) or through degradation by the MPS (a relatively fast process). Previously published data on free iodine pharmacokinetics and thyroid uptake rates were utilized to account for free label. A previous PK of free iodine showed biphasic kinetics with an alpha-phase half-life of 7 h, indicating the elimination half-life is unlikely to be faster than this value and was fixed in the model of free label (32). Iodine deconjugation was fixed to a rate of 0.000298 h^−1^ and occurred in every PBPK model compartment with labeled phage. Free iodine released by the MPS was fixed to a rate of 0.05 h^−1^ (33). Thyroid biological uptake half-life was fixed to 30 h based on literature reports of 16% iodine uptake by 6 h and 25% iodine uptake by 24 h (34). Though this thyroid uptake value is from humans, the slow uptake time relative to other kinetic processes would result in a negligible impact on the predicted %ID/g. Because most samples were obtained <24 h from administration, any free ^125^I that is present would be primarily due to *in vivo* catabolism of phage over other possible pathways.

Model parameters were estimated using SAEM in Monolix (2024R1, Lixoft) using a naïve pooled analysis (i.e., no random effects). After the structural model was finalized, phage size was assessed as a possible covariate on PBPK model parameters. It was not feasible to quantify inter-animal variability because concentration data were collected as a single terminal endpoint for each animal. However, to allow for efficient testing of size as a covariate on PBPK model parameters, a single SAEM iteration was performed using the final parameter estimates for the PBPK structural model, but assuming an interindividual variability of ω^2^ = 1 on each parameter of interest. This was done to generate empirical Bayesian estimates of each parameter for each mouse to quantify correlations between individual parameters and phage size or phage strain, which was used to prioritize the testing order of covariates, mirroring the COSSAC procedure. Model parameters that exhibited statistically significant correlations with phage size or phage strain were re-estimated with a phage size covariate effect using the naïve pooled analysis strategy used to determine the structural model. The process for assessing statistically significant effects of phage or phage size effects was performed on *K*_*p*_, *P*_*s*_, *A*_max_, *T*_up_, *T*_deg_, and *CL*_Active_. Final selection of covariates for inclusion was based on likelihood ratio testing at a significance level of α = 0.05.

### Sensitivity analysis

Local sensitivity analysis was performed to assess model changes in blood AUC in response to perturbations in *A*_max_, *CL*_active_, *K*_p,Crs_, *K*_p,LunKid_, *K*_p,LvrSpnSto_, *P*_*S*_, *T*_deg_, and *T*_up_. This was performed by running Monte Carlo simulations from the final model using a stepwise approach of modifying a single parameter value fivefold greater and lower than the estimated value. Mean change in AUC was calculated as:


(7) $$Normalized\;Change=\;\frac{AUC_{test}-AUC_{ref}}{AUC_{ref}}$$

Variance-based global sensitivity analysis (GSA) was performed using 2,000 samples of each parameter from a uniform distribution with limits that were fivefold greater and lower than the estimated parameter value. GSA was performed using the Sensitivity package (35) in R using the rank-based estimate of the first-order sensitivity indices (sobolrank) method. Ultimately, this approach allows for efficient assessment of global sensitivity to identify parameters, which can be better interrogated in future experiments.

### Interspecies scaling

To further qualify the final model, anatomical parameters for rats and humans were used to simulate phage concentration-time profiles after administration of phage. Tissue-resident macrophage concentrations were adjusted based on literature values. The full table of parameters used is in Table S1.

### External validation

The model was validated using data digitized from the literature (16, 36) using WebPlotDigitizer. Simulations were performed based on the listed dosing of phage in each study. For rats and humans, phage was assumed to be administered as a rapid 5 min push.

Phage PK in the Kim et al. study evaluated *Escherichia coli* phages, whereas the Li et al. study evaluated *Klebsiella pneumoniae* phages, challenging the final PBPK model to extrapolate across phage species. However, given limited phage PK data in the literature, this was the most robust method to qualify the final model. By evaluating model performance constructed on data generated on differentially sized *P. aeruginosa* phages, this external validation approach will provide information on the extrapolation potential of phage PK between phage genera.

In order to perform human simulations, estimated model parameters from murine studies were used, while human anatomical and tissue-resident macrophage concentrations were utilized. To simulate PFU per milliliter, doses were parameterized in PFU, and the untransformed macrophage capacity term was utilized (log_10_ PFU/100,000 cells). As such, this strategy minimized the number of model modifications needed to externally validate model estimates on the *Klebsiella* phage data in rodents and the *E. coli* phage data in humans. Observations versus predictions were quantitatively evaluated based on relative bias (rBias) and root mean square error (RMSE), as done previously (37).

### Clinical trial simulation

Simulation-based strategies were utilized to evaluate possible regimen structures of phage and provide a rational, data-driven basis for future trial design. Daily doses of 10^7^–10^12^ PFU were each tested with different administration schedules: single dose (×1), daily (Q24H), twice-daily (Q12H), and continuous infusion. Simulations were performed using phage dose units in PFU and were assumed to be independent of patient hematocrit and that phage did not distribute into the red blood cell space. Though phage pharmacodynamics are unquestionably important in identifying optimal regimens, standalone PK simulations are critical to designing future *in vivo* or clinical studies that will interrogate questions related to phage-bacterial interactions.

## RESULTS

### Whole-body distribution of phage and non-compartmental analysis

Phage concentrations in tissues varied greatly, with peak concentrations that ranged from 0.6 to 15 %ID/g between all tissues analyzed. Phage concentration declined within the first 1 h after administration, with a half-life of ≈ 0.3 h, then exhibited a terminal half-life of ≈ 8 h. The organs with the highest concentration of all three phages included the blood, lungs, liver, spleen, and kidneys. Interestingly, a median 18.0 %ID (SD = 6.4 %ID) of phage was discovered in the lumen of the stomach 1 to 4 h after penile vein administration (Fig. 2; Fig. S14).

A naïve pooled non-compartmental analysis was performed on median blood concentration values in each organ. Across all three phages, the median terminal half-life in blood was estimated as being 11.9 h, with individual half-lives for LUZ24, OMKO1, and PAML31 calculated as 10.3, 11.9, and 12.0 h, respectively. The observed AUC_0-last_ was 35.5 %ID*h/g after a single dose of phage across all studies. In blood, PAML31 exhibited a significantly larger AUC_0-last_ of 195 %ID*h/g, as compared to 35.5 %ID*h/g for LUZ24 or 33.1 %ID*h/g for OMKO1 (Fig. 2; Fig. S14)

### Physiologically based PK model of phage

**Fig 1:**
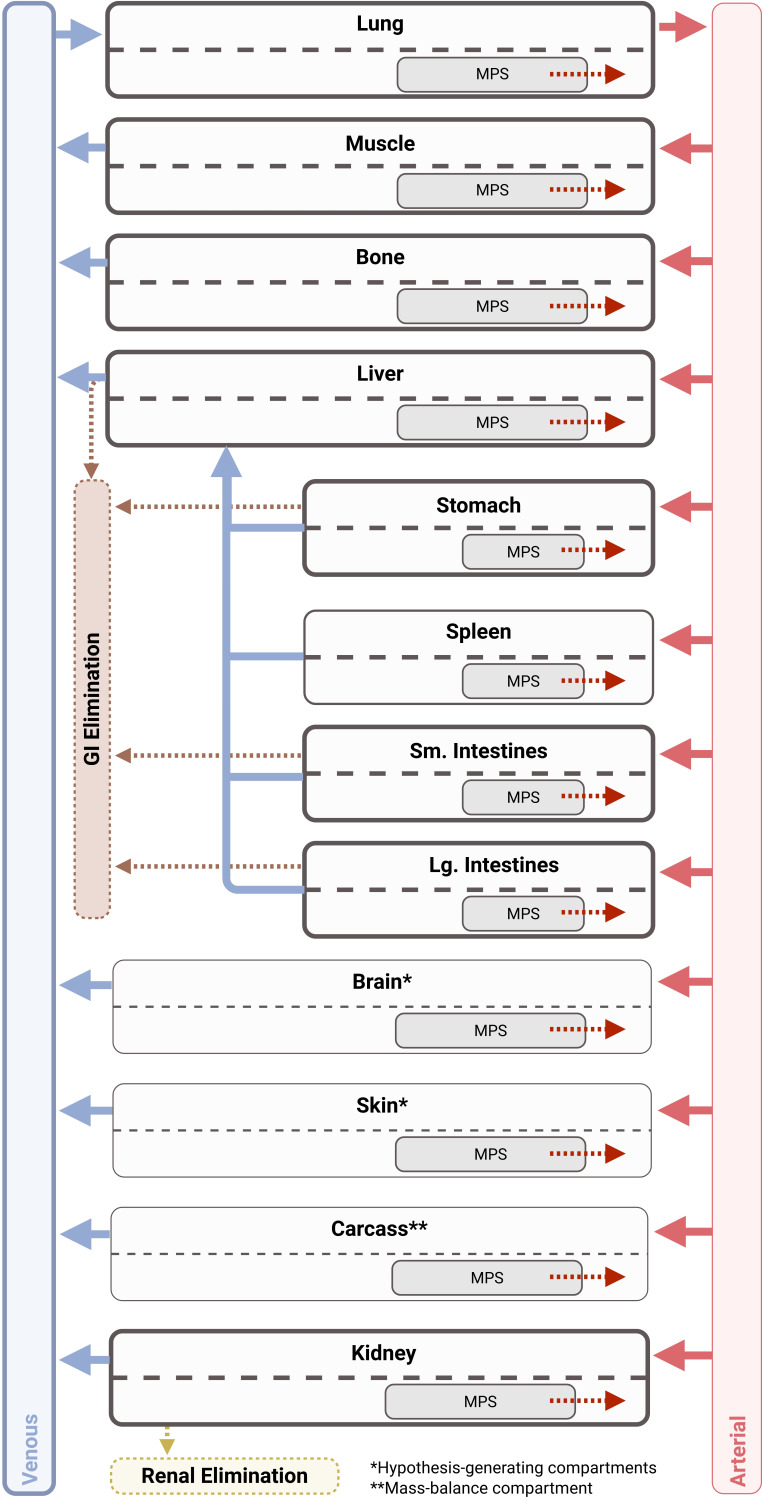
PBPK model structure. The final PBPK model included compartments describing the lung, muscle, bone, liver, stomach, spleen, small and large intestines, and kidney. Additional compartments were included for hypothesis-generating purposes (skin and brain), along with a compartment for mass balance (carcass). Phage clearance occurred through two pathways: RES-mediated clearance in each organ and active clearance at epithelial surfaces in the kidney, stomach, and intestines. Due to unique anatomical considerations, the liver receives dual input from the hepatic artery and the stomach, spleen, and intestines via the hepatic portal vein.

**Fig 2:**
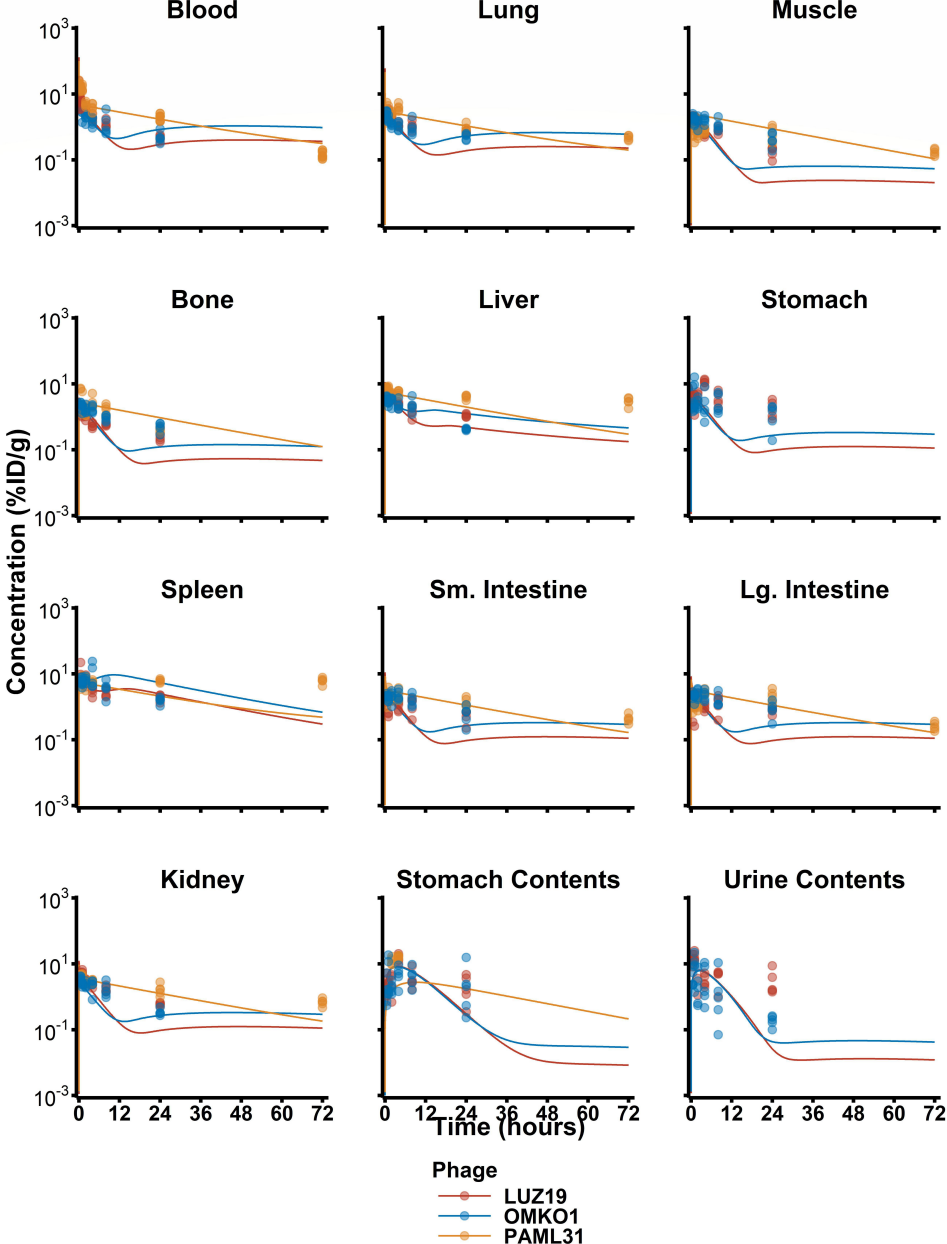
PBPK model *post hoc* fits. Raw data from animal replicates (dots) are presented along with PBPK model predictions (lines).

The final model structure is shown in Fig. 1, and model parameters are summarized in Table 1. Phage distribution was modeled as a permeability-limited process, where a whole-body permeability coefficient was simultaneously estimated, resulting in a final value of 0.0227. Tissue partitioning, *K*_*p*_, described the likelihood of phage being retained in each organ, and separate values were needed to describe high blood flow organs (i.e., lung and kidney) and gastrointestinal organs (i.e., liver, spleen, and stomach). (Fig. 2; Fig. S1 to S12) First-order active clearance from surfaces in the kidney (urinary excretion) and in the liver, stomach, and intestines (gastric excretion) was an additional elimination pathway and was estimated as 0.0145 L/h/kg. This parameter was, in part, informed by urine and stomach contents data. Additionally, MPS-mediated clearance was also estimated based on a degradation half-life in the MPS compartment within each tissue, which was estimated as 0.0301 h. Each tissue’s total capacity for phage was based on literature-derived values of tissue-resident phagocytes (see Table S1) and a literature-derived capacity of 10^3.81^ PFU/10^5^ phagocytes, based on cell uptake capacities determined *in vitro (*26). The uptake half-life (*T*_MPS,up_) was fixed at 0.001 with the assumption that endosomal degradation time of phage was rate-limiting. After performing a single expectation step (inter-animal variability fixed to ω = 1), correlations (>0.3) were detected between *CL*_Active_ and experiments conducted using the PAML-31 phage. The inclusion of a categorical covariate to describe a PAML-31-specific reduction in *CL*_Active_ resulted in a statistically significant improvement in the model and was estimated as being a 10.7-fold lower *CL*_Active_ for PAML-31 (0.00134 L/h/kg). Finally, stomach contents and urine measurements provided an opportunity to characterize potential phage elimination through alternative pathways. Estimation of gastric transit and urine transit half-lives was done as a mechanism to empirically account for ^125^I removal from these compartments (either as free label or labeled phage).

**TABLE 1:** Parameter estimates

Parameter	Definition	Units	Value	Linearization		
				R.S.E. (%)	P2.5	P97.5
*K*_p,LunKid_	Partition coefficient for high-flow organs (lung and kidney)	–^*c*^	0. 395	5.15	0.358	0.437434
*K*_p,LvrSpnSto_	Partition coefficient for Liver, spleen, and stomach	–	2.12e − 4	7.17	1.84e − 4	2.44e − 4
*K*_p,Crs_	Partition coefficient for rest of body	–	0.934	6.17	0.828	1.05
*P*_*s*_	Permeability coefficient	–	4.27e − 2	14.3	0.0324	0.0562
A_MPS_	MPS compartment capacity	Log_10_ (PFU/10^5^ cells)	3.81	Fixed^*a*^		
*T*_MPS,up_	Uptake half-life by MPS compartment	h	0.001	Fixed^*b*^		
*T*_MPS,deg_	Degradation rate phage in MPS compartment	h	4.37_E_ − 2	12.2	3.45e − 2	5.54e − 2
*CL*_Active_	Hypothetical “active” phage clearance at surface sites	L/h/kg	1.29_E_ − 2	5.10	1.16e − 2	1.42e − 2
θ_PAML31_	PAML-31 effect on CL_Active_	–	−1.94	5.73	−2.15	−1.72
*T*_Sto,out_	Gastric transit half-life	h	3.28	29.8	1.89	5.68
*T*_U,out_	Urine transit half-life	h	1.73	31.3	0.971	3.07
Covariate-adjusted parameters						
*CL*_Active,PAML_	Hypothetical “active” phage clearance at surface sites incorporating PAML-31 effect	L/h/kg	1.34_E_ − 3	0.205	0.0013	0.0014
Error model parameters						
σ_Pla_	Residual variance of plasma measure	%ID/g	4.28E + 00	6.00	3.80	4.81
σ_Lun_	Residual variance of lung measure	%ID/g	7.21E − 01	7.50	0.623	0.835
σ_Lvr_	Residual variance of liver measure	%ID/g	1.23E + 00	7.50	1.06	1.42
σ_Spn_	Residual variance of spleen measure	%ID/g	3.26E + 00	7.50	2.82	3.78
σ_Smi_	Residual variance of sm. intest. measure	%ID/g	1.18E + 00	7.50	1.02	1.36
σ_Lgi_	Residual variance of lg. intest. measure	%ID/g	1.39E + 00	7.50	1.20	1.61
σ_Msc_	Residual variance of muscle measure	%ID/g	9.02E − 01	7.50	0.779	1.04
σ_Kid_	Residual variance of kidney measure	%ID/g	8.99E − 01	7.50	0.776	1.04
σ_Sto_	Residual variance of stomach measure	%ID/g	4.15E + 00	9.21	3.471	4.97
σ_Bon_	Residual variance of bone measure	%ID/g	1.14E + 00	7.50	0.986	1.32
σ_Stc_	Residual variance of stomach contents measure	%ID/g	6.53E + 00	8.51	5.54	7.71
σ_Urn_	Residual variance of urine measure	%ID/g	6.12E + 00	9.53	5.08	7.37

^*a*^ Bichet et al. (26).

^*b*^ Fixed for model identifiability; NB: *k*_rel_ set equal to *k*_up,max_.

^*c*^ –, unitless parameters.

**Fig 3:**
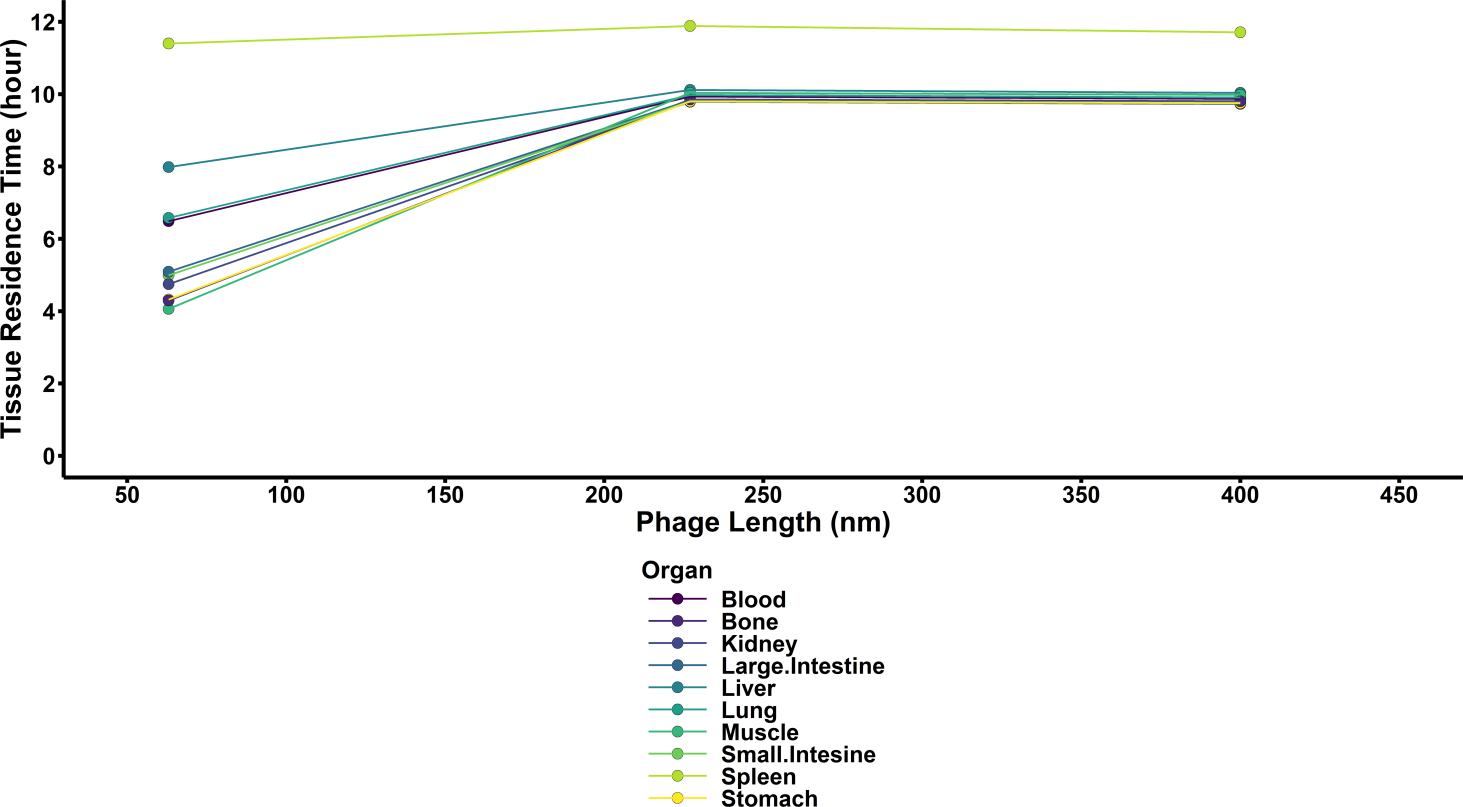
Tissue mean residence times of each phage. Tissue mean residence times were calculated numerically using the PBPK model as a ratio between the AUMC and AUC in each organ. Increased tissue residence times are associated with longer durations of phage residence in each organ and increase with size for most organs. Of note, the spleen had a more consistent residence time across all phage sizes.

Across all three phages in all tissues at 24 h, the spleen had the longest residence time (Fig. 3) of any organ (calculated as the ratio between the area under the first and zeroth moment curves using the PBPK model) at 10.7 h, with the liver having the second-longest residence time at 8.78 h. The muscle had the shortest residence time at 6.68 h.

### Sensitivity analysis

**Fig 4:**
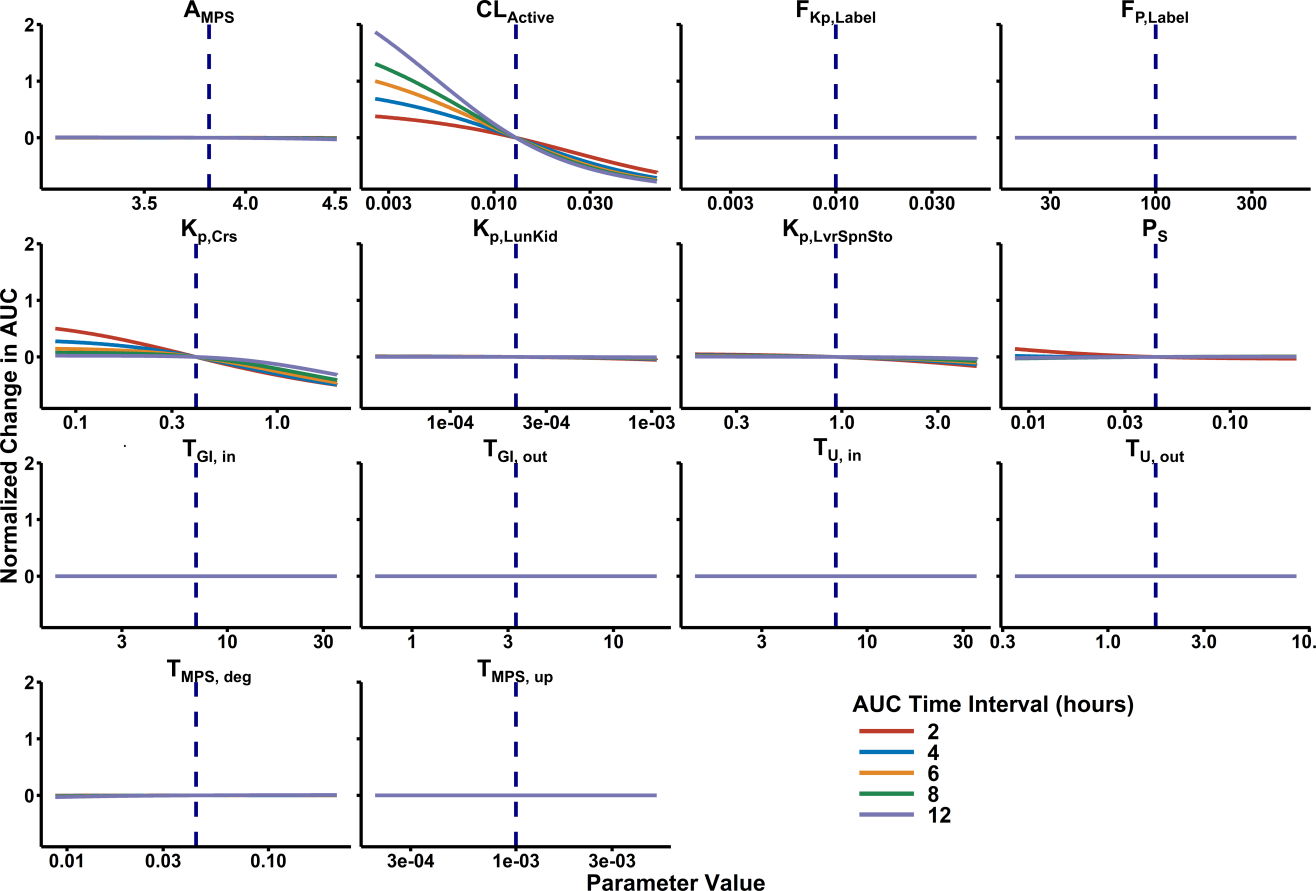
Local sensitivity analysis results. Local sensitivity analysis was performed by sequentially modifying each parameter to a maximum difference of ± 5-fold relative to the estimated value while all other parameters were fixed. In doing so, the magnitude of change in total blood exposure (i.e., area under the phage concentration-time curve) due to each PBPK model parameter can be evaluated. Sensitivity to each parameter was evaluated at 12, 24, and 48 h after phage administration to confirm minimal time-dependent effects.

The model was most sensitive (Fig. 4) to this hypothetical active clearance process, with a Sobol Rank Index of 0.907 (Bias = 0.00283, 95% CI 0.896–0.914). After *CL*_Active_, all other parameters had absolute Sobol Rank Indices ≤0.0330 (see Fig. S13). The next most sensitive parameters were *K*_*p*,Crs_ and *CL*_Active_. Of note, *A*_MPS_ was sampled on a log_10_ scale, which provides a significantly larger search space than the other parameters.

### Scaling to humans

**Fig 5:**
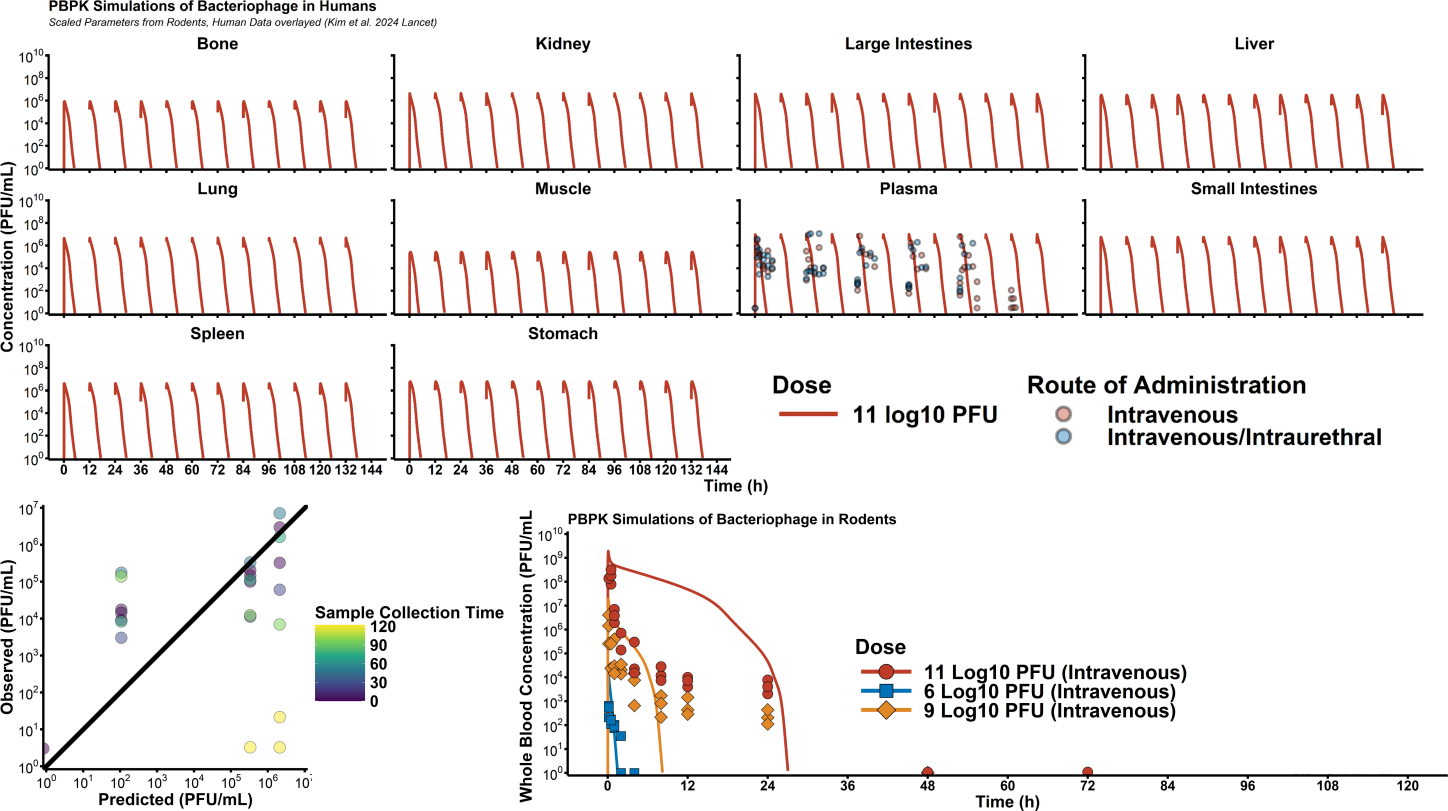
Human simulations and comparison to clinical data. To externally validate the PBPK model, we digitized rodent data using *Pseudomonas* phage øPEV20 (16, CMI) and CRISPR-enhanced *E. coli* phage LBP-EC01 (23, Lancet). For human simulations, observed clinical data were compared to model predictions, and rBias and rRMSE were calculated to assess performance. Rodent predictions at the highest dose level were significantly biased, likely due to model misspecification of the saturable clearance processes.

Human parameters are outlined in Table S1, with sources. Active clearance was allometrically scaled by body mass, assuming a human median mass of 70 kg. Simulation of 11 log_10_ PFU intravenously every 12 h (Q12H) showed reasonable agreement (Fig. 5; Fig. S15). Poor predictions primarily occurred at digitized data after 72 h. Overall, the rBias in the simulation predictions was −106%, with an rRMSE of 13.4%. When only evaluating the first 72 h of data, rBias increased to −121%, whereas rRMSE decreased to 9.98%. When attempting to simulate the PK of *Klebsiella* phages in rats, there was a significant overprediction of the highest 10^11^ PFU dose of phage, whereas the other two dose groups (10^9^ and 10^6^ PFU) were more accurately predicted.

**Fig 6:**
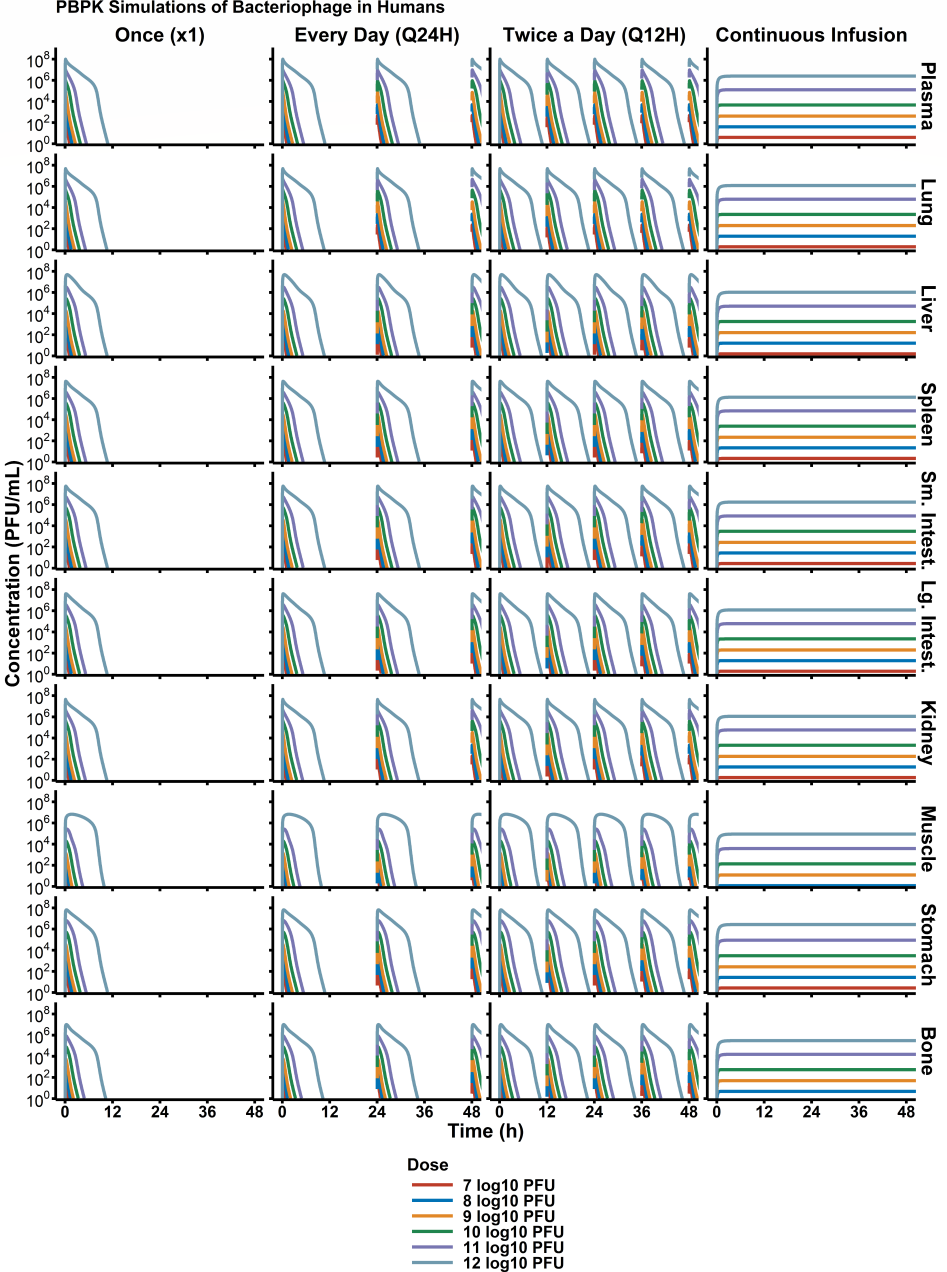
Simulated trial of phage PK in humans. To design first-in-human phage dosage regimens, simulation-based strategies are critical. Simulations of different dose levels and administration schedules (e.g., once, Q24H, Q12H) provided a robust understanding of expected phage concentrations in each organ. Overall, due to phage’s short half-life, continuous infusion reached steady state levels within 3 hours. With additional pharmacodynamic data, phage dosages can then be optimized to target specific, therapeutic concentrations in infection sites of interest.

Monte Carlo simulations (Fig. 6) predicted rapid elimination of phage in humans, resulting in phage blood concentrations being lower than 10^2^ PFU/mL (limit of quantification by plaque assay) by 12 h. As such, multi-dose regimens and continuous infusion regimens were the only strategies that allowed continuously detectable phage concentrations. Evaluation of different dose levels showed that at a maximum dose of 10^12^ PFU, phage concentrations are expected to be approximately 10^7^ PFU/g.

## DISCUSSION

Here, using PK data derived from nuclear imaging studies of three phages administered intravenously, we have developed a physiologically based model of phage PK. These data represent the first rigorous preclinical assessment of phage PK utilizing contemporary pharmacometric approaches amenable to both preclinical and clinical study design. This work innovates on previous studies of phage PK which were forced to rely upon data generated using laborious microbiological methods at sparse timepoints and analyzed using non-compartmental strategies. In doing so, we were able to establish a clinically translatable framework to study phage PK in preclinical animal models and better hypothesize clinical dosage regimens for use in humans.

Radionuclide labeling of pharmaceuticals is a longstanding strategy to quantify PK. Our use of a Sulfo-SHPP bi-functional conjugate for ^125^I labeling of phage capsid proteins had multiple advantages: (i) a protected aromatic ring to which oxidized ^125^I iodide ion is bound, (ii) an NHS ester which under physiologic conditions readily forms stable peptide (amide, covalent) bonds with accessible terminal amines of a protein, and (iii) is water-soluble at a neutral pH. To achieve this, we used a two-step method for phage radiolabeling by first oxidatively attaching ^125^I to Sulfo-SHPP using Iodobeads, which were then removed before adding to phage for a brief conjugation reaction. Critically, this two-step approach prevents oxidative damage and over-modification of phage to preserve structural integrity and infectivity.

^125^I-SHPP-labeled phage is highly resistant to spontaneous or enzymatic dehalogenation or proteolysis *in vivo* and ideal for PK/PD studies in preclinical models. In leveraging these radiolabeled phages in PK studies, sufficient data on phage concentrations in each tissue over time can be generated to inform a PBPK model of phage to more fully characterize phage distribution and elimination. Because phage catabolism is a major route of elimination, the final model implemented mass balance assumptions to account for ^125^I-phage being catabolized into ^125^I byproduct. This assumption could be violated by phage catabolism in other tissue sites or by other immune cells. Future PBPK modeling approaches may need to consider tissue-specific or cell type-specific clearance routes depending on the extent of impact for each route.

Regarding phage distribution, phage transport across cellular barriers is an area of active research. Historically, oral bioavailability of biologics and biotic agents is assumed to be negligible due to the combined effects of drug due to low pH, digestive enzymes, and low intestinal wall permeability. However, oral, intraurethral, or inhalational administration of phage has been shown to result in detectable blood concentrations of phage *in vivo* despite unclear mechanisms of phage transport across cellular barriers (36, 38). Both urine and stomach contents data were used to estimate the relative rate of phage entry and loss in each compartment, which limited mechanistic interpretability. However, the estimated rates of entry and loss support the need to expand our understanding of phage transit through cellular barriers. This study was also limited by the single terminal sampling and whole organ concentration measurements, which limited evaluation of distribution at the tissue and cellular levels. Future radiolabeling studies should consider obtaining spatial data on phage distribution, which would be best supplemented with live-cell imaging of phage interactions with mammalian cells, as done previously (26, 27).

In our study, differences in tissue mean residence time (Fig. 3) suggest tissue-specific and size-dependent trends, except for the spleen. The absence of discernible differences in the spleen or with LUZ24 mean residence time reflects macrophage-mediated clearance as a dominant elimination pathway. Our analytical approach was critical to being able to assess both phage-specific and size-dependent effects. Though size dependency was not estimated with statistical significance, this is likely due to only having three different covariate levels (63 nm, 227 nm, and 400 nm). Future studies should consider the influence of alternative biophysical measurements, such as hydrodynamic radius, capsid structure, gross morphology, etc. Additionally, this study confirms the ongoing utility of radiolabeling approaches to quantifying tissue concentrations of phages (29). Despite the lack of statistically significant differences associated with phage size, our findings suggest that size-dependent effects may be relevant (39, 40).

Recent work by Bichet and colleagues probed the question of transport of phage across cellular barriers and interestingly found a preferential transport direction of apical-to-basolateral sides in a transwell assay (26), which would ordinarily be seen as part of cellular uptake transport. The current assumptions supporting the PBPK model’s maximum phage capacity per macrophage may be mistaken, which is supported by slight misprediction of rodent data. This assumption was based on approximate calculations of digitized data that were generated for other purposes (see Supplemental material)*.* Thus, future studies should probe three key areas of phage cellular transport: rate, capacity, and directionality across different endothelial cells as an important determinant of distribution. Across all three areas, phage-determining effects should also be evaluated (e.g., phage size or morphology).

In our model, we preliminarily captured this cell-mediated clearance through the estimation of an empirical, first-order clearance at epithelial barriers (41). Unsurprisingly, this active clearance parameter was the most sensitive model parameter (e.g., Fig. 4), further highlighting the need to better understand phage clearance due to phage interaction with mammalian host cells. The statistically significant covariate effect estimated due to PAML-31-1 could represent potential phage-specific influences that might be better evaluated using *in vitro* phage-mammalian cell host assays. This shows the utility of this approach in identifying phage-specific effects, but it would be preferred to incorporate more generalizable phage properties. In sum, these findings highlight the continuing need for a more comprehensive understanding of phage-specific effects on clearance and interactions with mammalian cells.

This report highlights the comparative advantages of nuclear imaging-based approaches for phage PK studies. In comparison to conventional PK studies performed using time-consuming and labor-intensive traditional methods (qPCR, plaque assays), we found nuclear imaging of radiolabeled phages to be far more efficient. Nuclear medicine approaches will likely serve as an important tool for evaluating phage absorption, distribution, catabolism, and elimination in the absence of infection to provide a baseline for “ground truth” in phage disposition and elimination characteristics without the confounding effects of phage self-replication.

Simulation strategies were performed without regard for the potential for urinary phage to re-enter circulation and affect the observed PK. Though there is limited biological evidence for drugs re-entering systemic circulation from the bladder, the unique distribution properties of phages could require revisiting this commonly held assumption. Regardless, this assumption was still justified considering the extraordinarily high dosing level implemented (10^11^ PFU) in the clinical study relative to the approximate 10^5^ CFU/mL bacterial burden for a UTI and the amount of phage that is likely to be produced from auto-dosing from such concentrations.

Moving forward, PK studies of phage should focus on evaluating the effects of phage size and structure on distribution and elimination, but other salient features of phage biology should also be evaluated. For example, nanoparticles for pharmaceutical development are often characterized for their zeta potential, which has been shown to influence both formulation stability and pharmacokinetics. Optimal zeta potential has been reported as being between −30 and + 30, which coincides with previous reports of phage’s anionic zeta potential (25, 42).

Ultimately, the future of phage therapy as an antibiotic class orthogonal to small-molecule antibiotics is incredibly promising in the context of contemporary drug development strategies used for other biotic agents. Our PBPK modeling approach with radiolabeled phages represents a critical methodological breakthrough that directly addresses the historical barriers to phage therapy implementation by providing quantitative, reliable pharmacokinetic data for rational dosing regimens. These findings not only inform optimal administration strategies for the studied phages against *Pseudomonas aeruginosa* but also establish a translational framework that can accelerate the clinical development of diverse phage therapies against the growing spectrum of multidrug-resistant pathogens. If realized, phage therapy could restore the antimicrobial armamentarium and provide clinicians with a valuable resource in treating highly drug-resistant bacterial pathogens.

## SUPPLEMENTAL MATERIAL

Supplemental material aac.01506-25-s0001.docx Tables S1 to S4; Fig. S1 to S15.

ASM does not own the copyrights to Supplemental Material that may be linked to, or accessed through, an article. The authors have granted ASM a non-exclusive, world-wide license to publish the Supplemental Material files. Please contact the corresponding author directly for reuse.
