# Supplementary material for: Whole-body distribution of three Pseudomonas phages characterized by a translational physiologically based pharmacokinetic model
Source: Antimicrob Agents Chemother. 2025 Dec 5;70(1):e01506-25. doi: 10.1128/aac.01506-25 (PMC12777566; doi:10.1128/aac.01506-25)
Supplement: Supplemental material — Tables S1 to S4; Fig. S1 to S15. [file aac.01506-25-s0001.docx]

**SUPPLEMENTAL MATERIALS**

**Whole-body Distribution of three *Pseudomonas* Phages Characterized by a translational Physiologically based Pharmacokinetic Model**

**Arne Echterhof^1,2^, Tejas Dharmaraj^1^, Patrick Blankenberg^1^, Bobby Targ^1^, Thomas D. Nguyen^3^, Paul L. Bollyky^1^, Nicholas M. Smith^3^ Francis Blankenberg^4^**

**1)** Division of Infectious Diseases and Geographic Medicine, Department of Medicine, Stanford University School of Medicine, Stanford, California, USA.

**2)** Institute of Medical Microbiology, University Hospital of Muenster, Muenster, Germany

**3)** Division of Clinical and Translational Therapeutics, School of Pharmacy & Pharmaceutical Sciences, University at Buffalo, Buffalo, New York, USA.

**4)** Division of Pediatric Radiology and Nuclear Medicine

**Table S1: Phage dose and mouse weights for each phage study**

| **Row Labels** | **n** | **Mean Mouse Weight (g)** | **SD Mouse Weight (g)** | **Mean Phage Dose (Log10 PFU)** |
| --- | --- | --- | --- | --- |
| LUZ24 | 54 | 27.2 | 3.73 | 10.7 |
| OMKO1 | 30 | 34.5 | 2.70 | 10.1 |
| PAML31 | 55 | 34.4 | 1.49 | 11.9 |

**Table S2. Calculation of mean phage capacity per cell across different cell types as digitized from Bichet and colleagues(1).**

The capacity term was calculated using the geometric mean of all digitized counts across all observations and cell types at 18 hours. The authors reported performing experiments in T25cm2 dishes. We assumed confluency of 10^5^ cells per cm^2^, while authors reported that phages were applied at a saturating condition of 10^9^ PFU/mL. Therefore, we estimated total cellular number of approximately 2.5•10^6^. Using all digitized plaque counts at 18 hours, the geometric means of intracellular phage was determined to be 1.65 •10^5^ PFU (conversion from PFU/mL based on the original author’s supplemental methods section which says final stock for enumeration was reconstituted in 1 mL). Meaning that we assumed 1.65 •10^5^ PFU obtained from 2.5•10^6^ cells. This is approximate to what is reported from ThermoFisher on confluency in T-25 flasks (<https://www.thermofisher.com/us/en/home/references/gibco-cell-culture-basics/cell-culture-protocols/cell-culture-useful-numbers.html>). This was then converted from PFU/cell to PFU/100,000 cells for modeling purposes.

|  | **Hi** | **Intermediate** | **Low** |
| --- | --- | --- | --- |
|  | 2.22E+05 | 1.40E+05 | 2.23E+06 |
|  | 2.22E+05 | 1.40E+05 | 2.22E+06 |
|  | 1.66E+05 | 1.87E+05 | 2.49E+06 |
|  | 1.18E+05 | 3.73E+05 | 8.36E+04 |
|  | 1.18E+05 | 4.18E+05 | 3.96E+04 |
|  | 5.58E+04 | 4.71E+05 | 2.96E+04 |
|  | 6.25E+04 | 1.05E+06 | 2.50E+04 |
|  | 4.69E+04 | 1.25E+06 | 1.18E+04 |
|  | 2.22E+04 | 1.57E+06 | 8.86E+03 |
|  |  |  |  |
| **Cell type geometric mean** | 9.06E+04 | 4.35E+05 | 1.14E+05 |
|  |  |  |  |
| **Overall geometric mean** | 1.65E+05 |  |  |
| **PFU/cell** | 6.59E-02 |  |  |
| **PFU/100,000 cell** | 6.59E+03 |  |  |
|  |  |  |  |
| **Log(PFU/100,000 cell)** | 3.81913 |  |  |

**Table S3: PBPK Model Parameters**

| **Parameter** | **Definition** | **Units** | **Mouse** | **Rat** | **Human** | **Citation** |
| --- | --- | --- | --- | --- | --- | --- |
| CO | Cardiac output | L/h/kg | 16.5 | 15 | 16.5 | (2-8) |
| BW | Body weight | Kg | 0.02 | 0.25 | 70 |  |
| F_Q_Brn_ | Fraction of CO to Brain | - | 0.033 | 0.02 | 0.114 |  |
| F_Q_Lvr_ | Fraction of CO to Liver | - | 0.021 | 0.021 | 0.046 |  |
| F_Q_Spn_ | Fraction of CO to Spleen | - | 0.011 | 0.0085 | 0.01375 |  |
| F_Q_SI_ | Fraction of CO to Sm. Intest. | - | 0.105 | 0.104 | 0.13575 |  |
| F_Q_LI_ | Fraction of CO to Lg. Intest. | - | 0.0175 | 0.036 | 0.022625 |  |
| F_Q_Kid_ | Fraction of CO to Kidney | - | 0.091 | 0.141 | 0.175 |  |
| F_Q_Msc_ | Fraction of CO to muscle | - | 0.159 | 0.278 | 0.191 |  |
| F_Q_Ski_ | Fraction of CO to Skin | - | 0.058 | 0.058 | 0.058 |  |
| F_Q_Bon_ | Fraction of CO to Bone | - | 0.0267 | 0.122 | 0.042 |  |
| F_Q_Sto_ | Fraction of CO to Stomach | - | 0.017625 | 0.013 | 0.022625 |  |
| F_V_Lun_ | Fraction of BW as Lung Weight | - | 0.0073 | 0.005 | 0.008 |  |
| F_V_Brn_ | Fraction of BW as Brain | - | 0.0165 | 0.00057 | 0.02 |  |
| F_V_Lvr_ | Fraction of BW as Liver | - | 0.0549 | 0.0366 | 0.0257 |  |
| F_V_Spn_ | Fraction of BW as Lung | - | 0.005 | 0.002 | 0.00257 |  |
| F_V_SI_ | Fraction of BW as Sm. Intest | - | 0.0253 | 0.014 | 0.0091 |  |
| F_V_LI_ | Fraction of BW as Lg. Intest | - | 0.0109 | 0.0084 | 0.0053 |  |
| F_V_Kid_ | Fraction of BW as Kidney | - | 0.0167 | 0.0073 | 0.0044 |  |
| F_V_Msc_ | Fraction of BW as Muscle | - | 0.384 | 0.4043 | 0.4 |  |
| F_V_Ski_ | Fraction of BW as Skin | - | 0.1653 | 0.1903 | 0.0371 |  |
| F_V_Mar_ | Fraction of BW as Bone | - | 0.03 | 0.02 | 0.02 |  |
| Fr_V_Sto | Fraction of BW as Stomach | - | 0.006 | 0.0046 | 0.0021 |  |
| F_V_Pla_ | Fraction of BW as Blood | - | 0.029029 | 0.074 | 0.079 |  |
| F_V_Lun_Bld_ | Fraction of Lung that is Blood | - | 0.5 | 0.36 | 0.3867 |  |
| F_V_Brn_Bld_ | Fraction of Brain that is Blood | - | 0.03 | 0.03 | 0.04 |  |
| F_V_Lvr_Bld_ | Fraction of Liver that is Blood | - | 0.31 | 0.21 | 0.11 |  |
| F_V_Spn_Bld_ | Fraction of Spleen that is Blood | - | 0.17 | 0.22 | 0.3 |  |
| F_V_SI_Bld_ | Fraction of Sm. Intest. that is Blood | - | 0.24 | 0.3 | 0.3 |  |
| F_V_LI_Bld_ | Fraction of Lg. Intest. that is Blood | - | 0.24 | 0.3 | 0.3 |  |
| F_V_Kid_Bld_ | Fraction of Kidney that is blood | - | 0.24 | 0.16 | 0.36 |  |
| F_V_Msc_Bld_ | Fraction of Muscle that is blood | - | 0.04 | 0.04 | 0.01 |  |
| F_V_Ski_Bld_ | Fraction of Skin that is blood | - | 0.03 | 0.02 | 0.08 |  |
| F_V_Crs_Bld_ | Fraction of Carcass that is blood | - | 0.04 | 0.04 | 0.01 |  |
| F_V_Bon_Bld_ | Fraction of Bone that is blood | - | 0.1 | 0.04 | 0.04 |  |
| F_V_Sto_Bld_ | Fraction of stomach that is blood | - | 0.24 | 0.3 | 0.3 |  |
| M_Pla_ | Phagocytes in Blood | cells/g | 180000 | 14300 | 4170000 | (9-11) |
| M_Lun_ | Phagocytes in Lung | cells/g | 1710000 | 9210000 | 24800000 |  |
| M_Brn_ | Phagocytes in Brain | cells/g | 180000 | 390000 | 8400000 |  |
| M_Lvr_ | Phagocytes in Liver | cells/g | 1710000 | 2.70E+07 | 21800000 |  |
| M_Spn_ | Phagocytes in Spleen | cells/g | 1710000 | 2.28E+08 | 1.18E+08 |  |
| M_SI_ | Phagocytes in Sm. Intest. | cells/g | 180000 | 60000 | 1710000 |  |
| M_LI_ | Phagocytes in Lg. Intest | cells/g | 180000 | 15000 | 11200000 |  |
| M_Kid_ | Phagocytes in Kidney | cells/g | 180000 | 390000 | 2210000 |  |
| M_Crs_ | Phagocytes in Carcass | cells/g | 180000 | 6350000 | 2630000 |  |
| M_Msc_ | Phagocytes in Muscle | cells/g | 180000 | 40000 | 773000 |  |
| M_Ski_ | Phagocytes in Skin | cells/g | 180000 | 40000 | 3910000 |  |
| M_Bon_ | Phagocytes in Bone | cells/g | 180000 | 14900000 | 45300000 |  |
| M_Sto_ | Phagocytes in Stomach | cells/g | 180000 | 6350000 | 936000 |  |

**Equations**

Lung (Lun)

$$\frac{dC_{Lun,V}}{dt}\cdot V_{Lun,V}=Q_{Lun}\cdot\left( C_{a}\left( t \right)-C_{Lun,V}\left( t \right) \right)-P_{S}\cdot Q_{Lun}\cdot\left( C_{Lun,V}\left( t \right)-\frac{C_{Lun,int}\left( t \right)}{K_{p,LunKid}} \right)$$

$$\frac{{dC}_{Lun,int}}{dt}\cdot V_{Lun,int}=P_{S}\cdot Q_{Lun}\cdot\left( C_{Lun,V}\left( t \right)-\frac{C_{Lun,int}\left( t \right)}{K_{p,LunKid}} \right)-k_{Lun,up}\left( t \right)\cdot C_{Lun,int}\left( t \right)\cdot V_{Lun,int}+k_{rel}\cdot A_{Lun,res}\left( t \right)$$

$$\frac{{dA}_{Lun,res}}{dt}=k_{Lun,up}\left( t \right)\cdot C_{Lun,int}\left( t \right)\cdot V_{Lun,int}-k_{rel}\cdot A_{Lun,res}\left( t \right)-k_{deg}\cdot A_{Lun,res}\left( t \right)$$

$$k_{Lun,up}\left( t \right)=k_{up,max}\cdot\left( 1-\frac{A_{Lun,res}\left( t \right)}{A_{Lun,max}} \right)$$

$$A_{Lun,max}=M_{Lun}\cdot V_{Lun}\cdot\left( \frac{A_{RES}}{{10}^{5}} \right)$$

Muscle (Msc)

$$\frac{dC_{Msc,V}}{dt}\cdot V_{Msc,V}=Q_{Msc}\cdot\left( C_{a}\left( t \right)-C_{Msc,V}\left( t \right) \right)-P_{S}\cdot Q_{Msc}\cdot\left( C_{Msc,V}\left( t \right)-\frac{C_{Msc,int}\left( t \right)}{K_{p,Crs}} \right)$$

$$\frac{{dC}_{Msc,int}}{dt}\cdot V_{Msc,int}=P_{S}\cdot Q_{Msc}\cdot\left( C_{Msc,V}\left( t \right)-\frac{C_{Msc,int}\left( t \right)}{K_{p,Crs}} \right)-k_{Msc,up}\left( t \right)\cdot C_{Msc,int}\left( t \right)\cdot V_{Msc,int}+k_{rel}\cdot A_{Msc,res}\left( t \right)$$

$$\frac{{dA}_{Msc,res}}{dt}=k_{Msc,up}\left( t \right)\cdot C_{Msc,int}\left( t \right)\cdot V_{Msc,int}-k_{rel}\cdot A_{Msc,res}\left( t \right)-k_{deg}\cdot A_{Msc,res}\left( t \right)$$

$$k_{Msc,up}\left( t \right)=k_{up,max}\cdot\left( 1-\frac{A_{MSc,res}\left( t \right)}{A_{Msc,max}} \right)$$

$$A_{Msc,max}=M_{Msc}\cdot V_{Msc}\cdot\left( \frac{A_{RES}}{{10}^{5}} \right)$$

Bone (Bon)

$$\frac{dC_{Bon,V}}{dt}\cdot V_{Bon,V}=Q_{Bon}\cdot\left( C_{a}\left( t \right)-C_{Bon,V}\left( t \right) \right)-P_{S}\cdot Q_{Bon}\cdot\left( C_{Bon,V}\left( t \right)-\frac{C_{Bon,int}\left( t \right)}{K_{p,Crs}} \right)$$

$$\frac{{dC}_{Bon,int}}{dt}\cdot V_{Bon,int}=P_{S}\cdot Q_{Bon}\cdot\left( C_{Bon,V}\left( t \right)-\frac{C_{Bon,int}\left( t \right)}{K_{p,Crs}} \right)-k_{Bon,up}\left( t \right)\cdot C_{Bon,int}\left( t \right)\cdot V_{Bon,int}+k_{rel}\cdot A_{Bon,res}\left( t \right)$$

$$\frac{{dA}_{Bon,res}}{dt}=k_{Bon,up}\left( t \right)\cdot C_{Bon,int}\left( t \right)\cdot V_{Bon,int}-k_{rel}\cdot A_{Bon,res}\left( t \right)-k_{deg}\cdot A_{Bon,res}\left( t \right)$$

$$k_{Bon,up}\left( t \right)=k_{up,max}\cdot\left( 1-\frac{A_{Bon,res}\left( t \right)}{A_{Bon,max}} \right)$$

$$A_{Bon,max}=M_{Bon}\cdot V_{Bon}\cdot\left( \frac{A_{RES}}{{10}^{5}} \right)$$

Liver (Lvr)

$$\frac{dC_{Lvr,V}}{dt}\cdot V_{Lvr,V}=Q_{Lvr}\cdot C_{a}\left( t \right)-\left( Q_{Lvr}+Q_{Hpv} \right)\cdot C_{Lvr,V}\left( t \right)+Q_{Spn}\cdot C_{Spn,V}\left( t \right)+Q_{SI}\cdot C_{SI,V}\left( t \right)+Q_{LI}\cdot C_{LI,V}\left( t \right)+Q_{Sto}\cdot C_{Sto,V}\left( t \right)-P_{S}\cdot Q_{Lvr}\cdot\left( C_{Lvr,V}\left( t \right)-\frac{C_{Lvr,int}\left( t \right)}{K_{p,LvrSpnSto}} \right)-{CL}_{Active}\cdot C_{Lvr,V}\left( t \right)$$

$$Q_{Hpv}=Q_{Spn}+Q_{Sto}+Q_{SI}+Q_{LI}$$

$$\frac{{dC}_{Lvr,int}}{dt}\cdot V_{Lvr,int}=P_{S}\cdot Q_{Lvr}\cdot\left( C_{Lvr,V}\left( t \right)-\frac{C_{Lvr,int}\left( t \right)}{K_{p,LvrSpnSto}} \right)-k_{Lvr,up}\left( t \right)\cdot C_{Lvr,int}\left( t \right)\cdot V_{Lvr,int}+k_{rel}\cdot A_{Lvr,res}\left( t \right)$$

$$\frac{{dA}_{Lvr,res}}{dt}=k_{Lvr,up}\left( t \right)\cdot C_{Lvr,int}\left( t \right)\cdot V_{Lvr,int}-k_{rel}\cdot A_{Lvr,res}\left( t \right)-k_{deg}\cdot A_{Lvr,res}\left( t \right)$$

$$k_{Lvr,up}\left( t \right)=k_{up,max}\cdot\left( 1-\frac{A_{Lvr,res}\left( t \right)}{A_{Lvr,max}} \right)$$

$$A_{Lvr,max}=M_{Lvr}\cdot V_{Lvr}\cdot\left( \frac{A_{RES}}{{10}^{5}} \right)$$

Stomach (Sto)

$$\frac{dC_{Sto,V}}{dt}\cdot V_{Sto,V}=Q_{Sto}\cdot\left( C_{a}\left( t \right)-C_{Sto,V}\left( t \right) \right)-P_{S}\cdot Q_{Sto}\cdot\left( C_{Sto,V}\left( t \right)-\frac{C_{Sto,int}\left( t \right)}{K_{p,LvrSpnSto}} \right)-{CL}_{Active}\cdot C_{Sto,V}\left( t \right)$$

$$\frac{{dC}_{Sto,int}}{dt}\cdot V_{Sto,int}=P_{S}\cdot Q_{Sto}\cdot\left( C_{Sto,V}\left( t \right)-\frac{C_{Sto,int}\left( t \right)}{K_{p,LvrSpnSto}} \right)-k_{Sto,up}\left( t \right)\cdot C_{Sto,int}\left( t \right)\cdot V_{Sto,int}+k_{rel}\cdot A_{Sto,res}\left( t \right)$$

$$\frac{{dA}_{Sto,res}}{dt}=k_{Sto,up}\left( t \right)\cdot C_{Sto,int}\left( t \right)\cdot V_{Sto,int}-k_{rel}\cdot A_{Sto,res}\left( t \right)-k_{deg}\cdot A_{Sto,res}\left( t \right)$$

$$k_{Sto,up}\left( t \right)=k_{up,max}\cdot\left( 1-\frac{A_{Sto,res}\left( t \right)}{A_{Sto,max}} \right)$$

$$A_{Sto,max}=M_{Sto}\cdot V_{Sto}\cdot\left( \frac{A_{RES}}{{10}^{5}} \right)$$

Spleen (Spn)

$$\frac{dC_{Spn,V}}{dt}\cdot V_{Spn,V}=Q_{Spn}\cdot\left( C_{a}\left( t \right)-C_{Spn,V}\left( t \right) \right)-P_{S}\cdot Q_{Spn}\cdot\left( C_{Spn,V}\left( t \right)-\frac{C_{Spn,int}\left( t \right)}{K_{p,LvrSpnSto}} \right)$$

$$\frac{{dC}_{Spn,int}}{dt}\cdot V_{Spn,int}=P_{S}\cdot Q_{Spn}\cdot\left( C_{Spn,V}\left( t \right)-\frac{C_{Spn,int}\left( t \right)}{K_{p,LvrSpnSto}} \right)-k_{Spn,up}\left( t \right)\cdot C_{Spn,int}\left( t \right)\cdot V_{Spn,int}+k_{rel}\cdot A_{Spn,res}\left( t \right)$$

$$\frac{{dA}_{Spn,res}}{dt}=k_{Spn,up}\left( t \right)\cdot C_{Spn,int}\left( t \right)\cdot V_{Spn,int}-k_{rel}\cdot A_{Spn,res}\left( t \right)-k_{deg}\cdot A_{Spn,res}\left( t \right)$$

$$k_{Spn,up}\left( t \right)=k_{up,max}\cdot\left( 1-\frac{A_{Spn,res}\left( t \right)}{A_{Spn,max}} \right)$$

$$A_{Spn,max}=M_{Spn}\cdot V_{Spn}\cdot\left( \frac{A_{RES}}{{10}^{5}} \right)$$

Small Intestine (SI)

$$\frac{dC_{SI,V}}{dt}\cdot V_{SI,V}=Q_{SI}\cdot\left( C_{a}\left( t \right)-C_{SI,V}\left( t \right) \right)-P_{S}\cdot Q_{SI}\cdot\left( C_{SI,V}\left( t \right)-\frac{C_{SI,int}\left( t \right)}{K_{p,Crs}} \right)-{CL}_{Active}\cdot C_{SI,V}\left( t \right)$$

$$\frac{{dC}_{SI,int}}{dt}\cdot V_{SI,int}=P_{S}\cdot Q_{SI}\cdot\left( C_{SI,V}\left( t \right)-\frac{C_{SI,int}\left( t \right)}{K_{p,Crs}} \right)-k_{SI,up}\left( t \right)\cdot C_{SI,int}\left( t \right)\cdot V_{SI,int}+k_{rel}\cdot A_{SI,res}\left( t \right)$$

$$\frac{{dA}_{SI,res}}{dt}=k_{SI,up}\left( t \right)\cdot C_{SI,int}\left( t \right)\cdot V_{SI,int}-k_{rel}\cdot A_{SI,res}\left( t \right)-k_{deg}\cdot A_{SI,res}\left( t \right)$$

$$k_{Spn,up}\left( t \right)=k_{up,max}\cdot\left( 1-\frac{A_{Spn,res}\left( t \right)}{A_{Spn,max}} \right)$$

$$A_{Spn,max}=M_{Spn}\cdot V_{Spn}\cdot\left( \frac{A_{RES}}{{10}^{5}} \right)$$

Large Intestine (LI)

$$\frac{dC_{LI,V}}{dt}\cdot V_{LI,V}=Q_{LI}\cdot\left( C_{a}\left( t \right)-C_{LI,V}\left( t \right) \right)-P_{S}\cdot Q_{LI}\cdot\left( C_{LI,V}\left( t \right)-\frac{C_{LI,int}\left( t \right)}{K_{p,Crs}} \right)-{CL}_{Active}\cdot C_{LI,V}\left( t \right)$$

$$\frac{{dC}_{LI,int}}{dt}\cdot V_{LI,int}=P_{S}\cdot Q_{LI}\cdot\left( C_{LI,V}\left( t \right)-\frac{C_{LI,int}\left( t \right)}{K_{p,Crs}} \right)-k_{LI,up}\left( t \right)\cdot C_{LI,int}\left( t \right)\cdot V_{LI,int}+k_{rel}\cdot A_{LI,res}\left( t \right)$$

$$\frac{{dA}_{LI,res}}{dt}=k_{LI,up}\left( t \right)\cdot C_{LI,int}\left( t \right)\cdot V_{LI,int}-k_{rel}\cdot A_{LI,res}\left( t \right)-k_{deg}\cdot A_{LI,res}\left( t \right)$$

$$k_{LI,up}\left( t \right)=k_{up,max}\cdot\left( 1-\frac{A_{LI,res}\left( t \right)}{A_{LI,max}} \right)$$

$$A_{LI,max}=M_{LI}\cdot V_{LI}\cdot\left( \frac{A_{RES}}{{10}^{5}} \right)$$

Brain (Brn)

$$\frac{{dC}_{Brn,V}}{dt}\cdot V_{Brn,V}=Q_{Brn}\cdot\left( C_{a}\left( t \right)-C_{Brn,V}\left( t \right) \right)-P_{S}\cdot Q_{Brn}\cdot\left( C_{Brn,V}\left( t \right)-\frac{C_{Brn,int}\left( t \right)}{K_{p,Crs}} \right)$$

$$\frac{{dC}_{Brn,int}}{dt}\cdot V_{Brn,int}=P_{S}\cdot Q_{Brn}\cdot\left( C_{Brn,V}\left( t \right)-\frac{C_{Brn,int}\left( t \right)}{K_{p,Crs}} \right)$$

Skin (Ski)

$$\frac{{dC}_{Ski,V}}{dt}\cdot V_{Ski,V}=Q_{Brn}\cdot\left( C_{a}\left( t \right)-C_{Ski,V}\left( t \right) \right)-P_{S}\cdot Q_{Ski}\cdot\left( C_{Ski,V}\left( t \right)-\frac{C_{Ski,int}\left( t \right)}{K_{p,Crs}} \right)$$

$$\frac{{dC}_{Ski,V}}{dt}\cdot V_{Ski,V}=P_{S}\cdot Q_{Ski}\cdot\left( C_{Ski,V}\left( t \right)-\frac{C_{Ski,int}\left( t \right)}{K_{p,Crs}} \right)-k_{Ski,up}\left( t \right)\cdot C_{Ski,int}\left( t \right)\cdot V_{Ski,int}+k_{rel}\cdot A_{Ski,res}\left( t \right)$$

$$\frac{{dA}_{Ski,res}}{dt}=k_{Ski,up}\left( t \right)\cdot C_{Ski,int}\left( t \right)\cdot V_{Ski,int}-k_{rel}\cdot A_{LI,res}\left( t \right)-k_{deg}\cdot A_{Ski,res}\left( t \right)$$

$$k_{Ski,up}\left( t \right)=k_{up,max}\cdot\left( 1-\frac{A_{Ski,res}\left( t \right)}{A_{Ski,max}} \right)$$

$$A_{Ski,max}=M_{Ski}\cdot V_{Ski}\cdot\left( \frac{A_{RES}}{{10}^{5}} \right)$$

Carcass (Crs)

$$\frac{{dC}_{Crs,V}}{dt}\cdot V_{Crs,V}=Q_{Crs}\cdot\left( C_{a}\left( t \right)-C_{Crs,V}\left( t \right) \right)-P_{S}\cdot Q_{Crs}\cdot\left( C_{Crs,V}\left( t \right)-\frac{C_{Crs,int}\left( t \right)}{K_{p,Crs}} \right)$$

$$\frac{{dC}_{Crs,V}}{dt}\cdot V_{Crs,V}=P_{S}\cdot Q_{Crs}\cdot\left( C_{Crs,V}\left( t \right)-\frac{C_{Crs,int}\left( t \right)}{K_{p,Crs}} \right)-k_{Crs,up}\left( t \right)\cdot C_{Crs,int}\left( t \right)\cdot V_{Crs,int}+k_{rel}\cdot A_{Crs,res}\left( t \right)$$

$$\frac{{dA}_{Crs,res}}{dt}=k_{Crs,up}\left( t \right)\cdot C_{Crs,int}\left( t \right)\cdot V_{Crs,int}-k_{rel}\cdot A_{Crs,res}\left( t \right)-k_{deg}\cdot A_{Crs,res}\left( t \right)$$

$$k_{Crs,up}\left( t \right)=k_{up,max}\cdot\left( 1-\frac{A_{Crs,res}\left( t \right)}{A_{Crs,max}} \right)$$

$$A_{Crs,max}=M_{Crs}\cdot V_{Crs}\cdot\left( \frac{A_{RES}}{{10}^{5}} \right)$$

Kidney (Kid)

$$\frac{{dC}_{Kid,V}}{dt}\cdot V_{Kid,V}=Q_{Kid}\cdot\left( C_{a}\left( t \right)-C_{Kid,V}\left( t \right) \right)-P_{S}\cdot Q_{Kid}\cdot\left( C_{Kid,V}\left( t \right)-\frac{C_{Kid,int}\left( t \right)}{K_{p,LunKid}} \right)-{CL}_{Active}\cdot C_{Kid,V}\left( t \right)$$

$$\frac{{dC}_{Kid,V}}{dt}\cdot V_{Kid,V}=P_{S}\cdot Q_{Kid}\cdot\left( C_{Kid,V}\left( t \right)-\frac{C_{Kid,int}\left( t \right)}{K_{p,LunKid}} \right)-k_{Kid,up}\left( t \right)\cdot C_{Kid,int}\left( t \right)\cdot V_{Kid,int}+k_{rel}\cdot A_{Kid,res}\left( t \right)$$

$$\frac{{dA}_{Kid,res}}{dt}=k_{Kid,up}\left( t \right)\cdot C_{Kid,int}\left( t \right)\cdot V_{Kid,int}-k_{rel}\cdot A_{Kid,res}\left( t \right)-k_{deg}\cdot A_{Kid,res}\left( t \right)$$

$$\frac{{dA}_{Kid,Urine}}{dt}={CL}_{Active}\cdot C_{Kid,V}\left( t \right)-k_{Kid,urine}\cdot A_{Kid,Urine}\left( t \right)$$

$$k_{Kid,up}\left( t \right)=k_{up,max}\cdot\left( 1-\frac{A_{Kid,res}\left( t \right)}{A_{Kid,max}} \right)$$

$$A_{Kid,max}=M_{Kid}\cdot V_{Kid}\cdot\left( \frac{A_{RES}}{{10}^{5}} \right)$$

Venous

$$\frac{dC_{\text{V}}}{dt}\cdot V_{\text{V}}=Q_{Brn}\cdot C_{Brn,v}\left( t \right)+\left( Q_{Lvr}+Q_{Hpv} \right)\cdot C_{Lvr,v}\left( t \right)+Q_{Kid}\cdot C_{Kid,v}\left( t \right)+Q_{Msc}\cdot C_{Msc,v}\left( t \right)+Q_{Ski}\cdot C_{Ski,v}\left( t \right)+Q_{Bon}\cdot C_{Bon,v}\left( t \right)+Q_{Crs}\cdot C_{Crs,v}\left( t \right)-Q_{Lun}\cdot C_{Lun,v}\left( t \right)-k_{V,up}\cdot C_{V}\left( t \right)\cdot V_{V}+k_{rel}\cdot A_{V,res}(t)$$

Note, in all cases $k_{rel}=k_{\text{up,max}}$, due to limited ability to differentiate the difference between degradation-limited and cell entry-limited kinetics. Degradation rate was estimated under the assumption it was the rate limiting process.

Figure S1: Observed versus predicted diagnostic plot of Blood measurements.


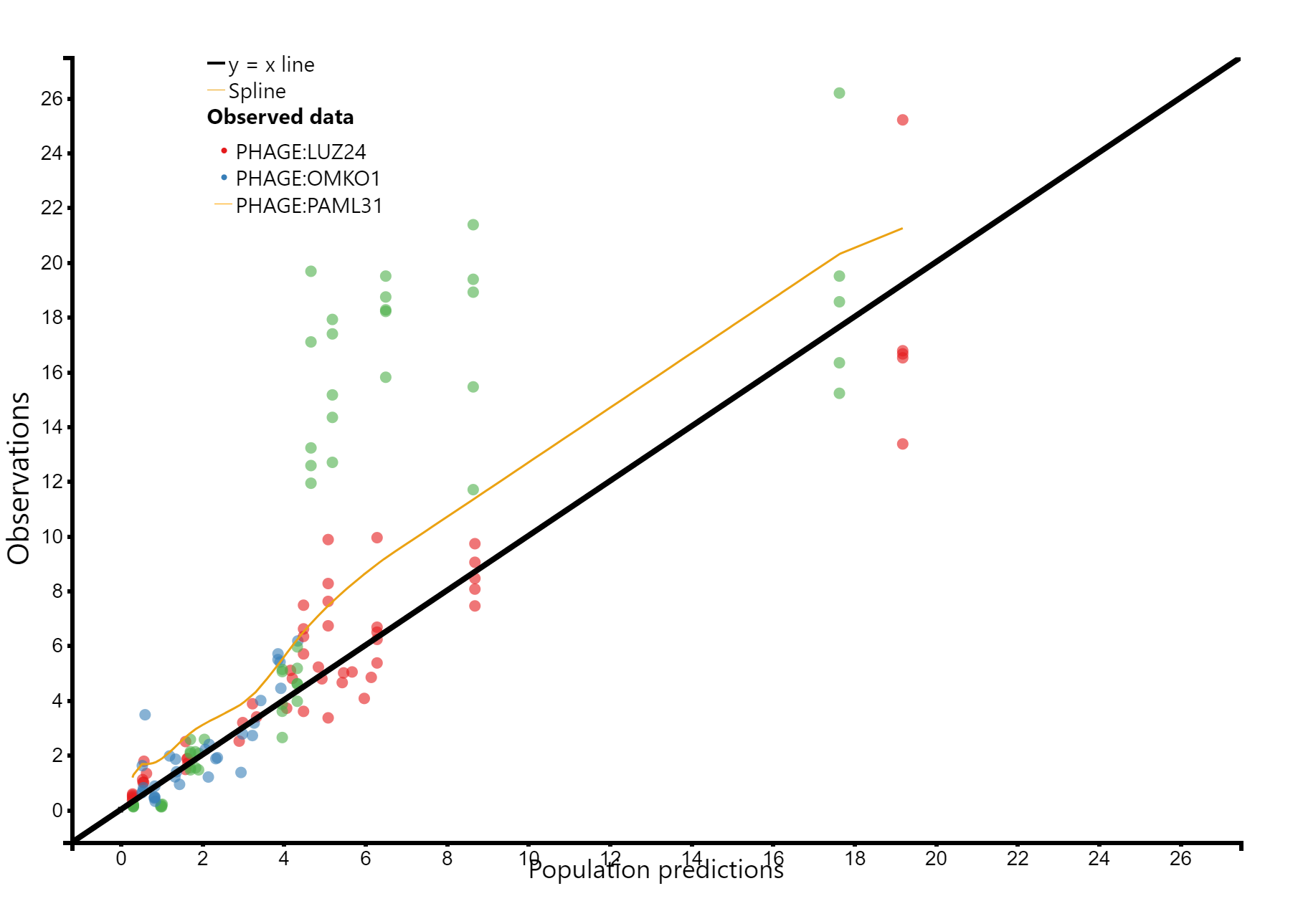


Figure S2: Observed versus predicted diagnostic plot of Bone measurements.


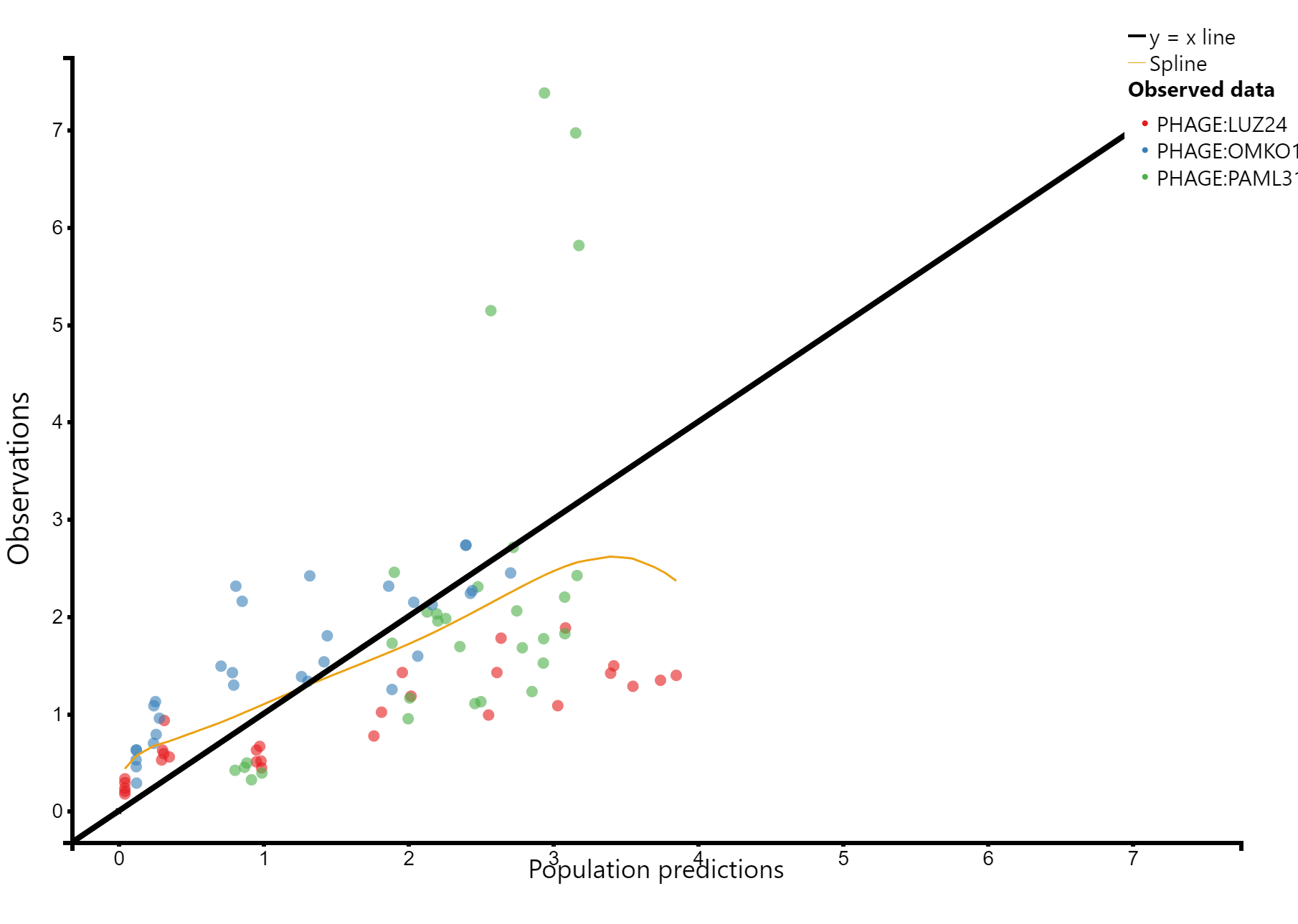


Figure S3: Observed versus predicted diagnostic plot of Kidney measurements.


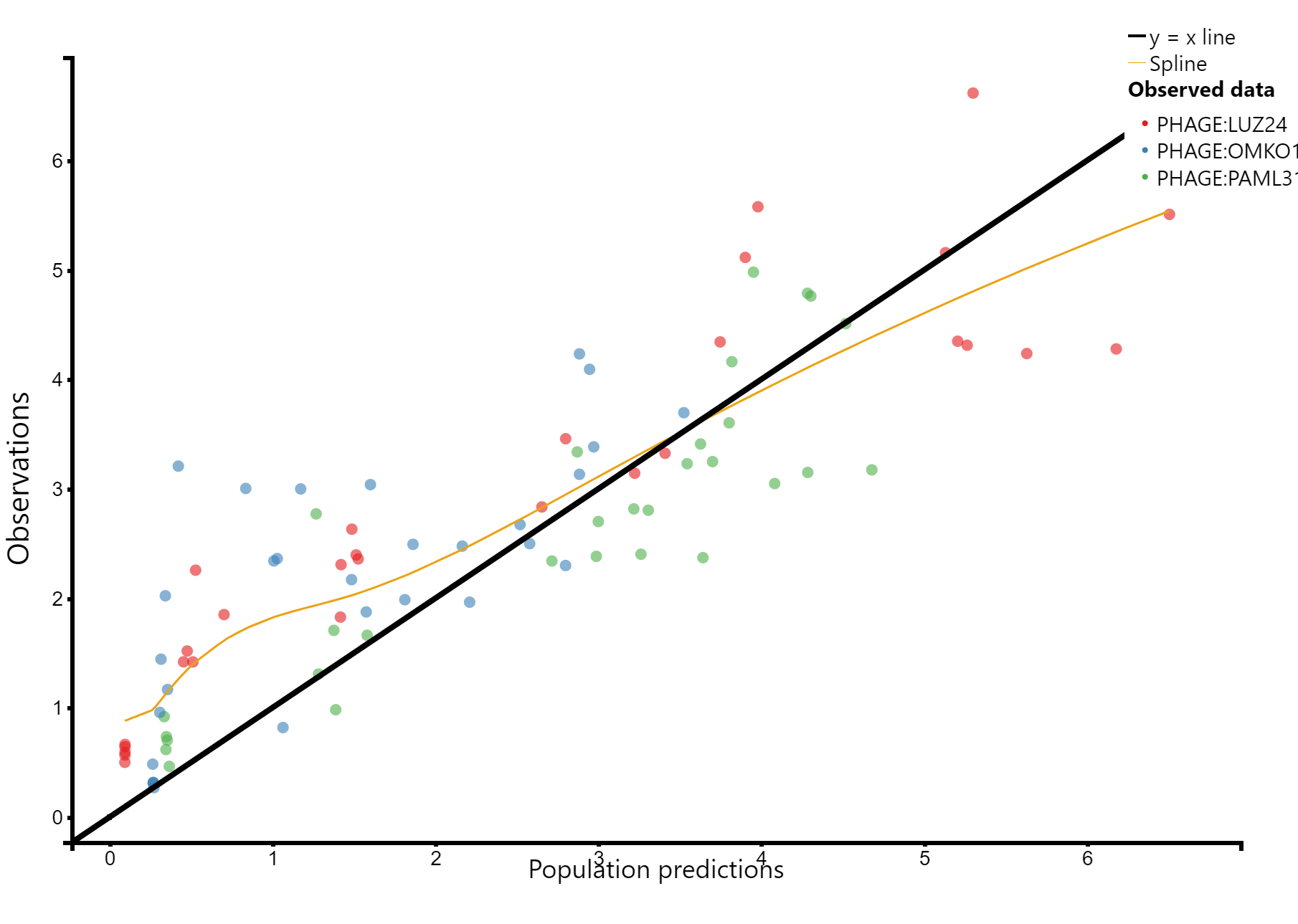


Figure S4: Observed versus predicted diagnostic plot of Lg. Intestines measurements.


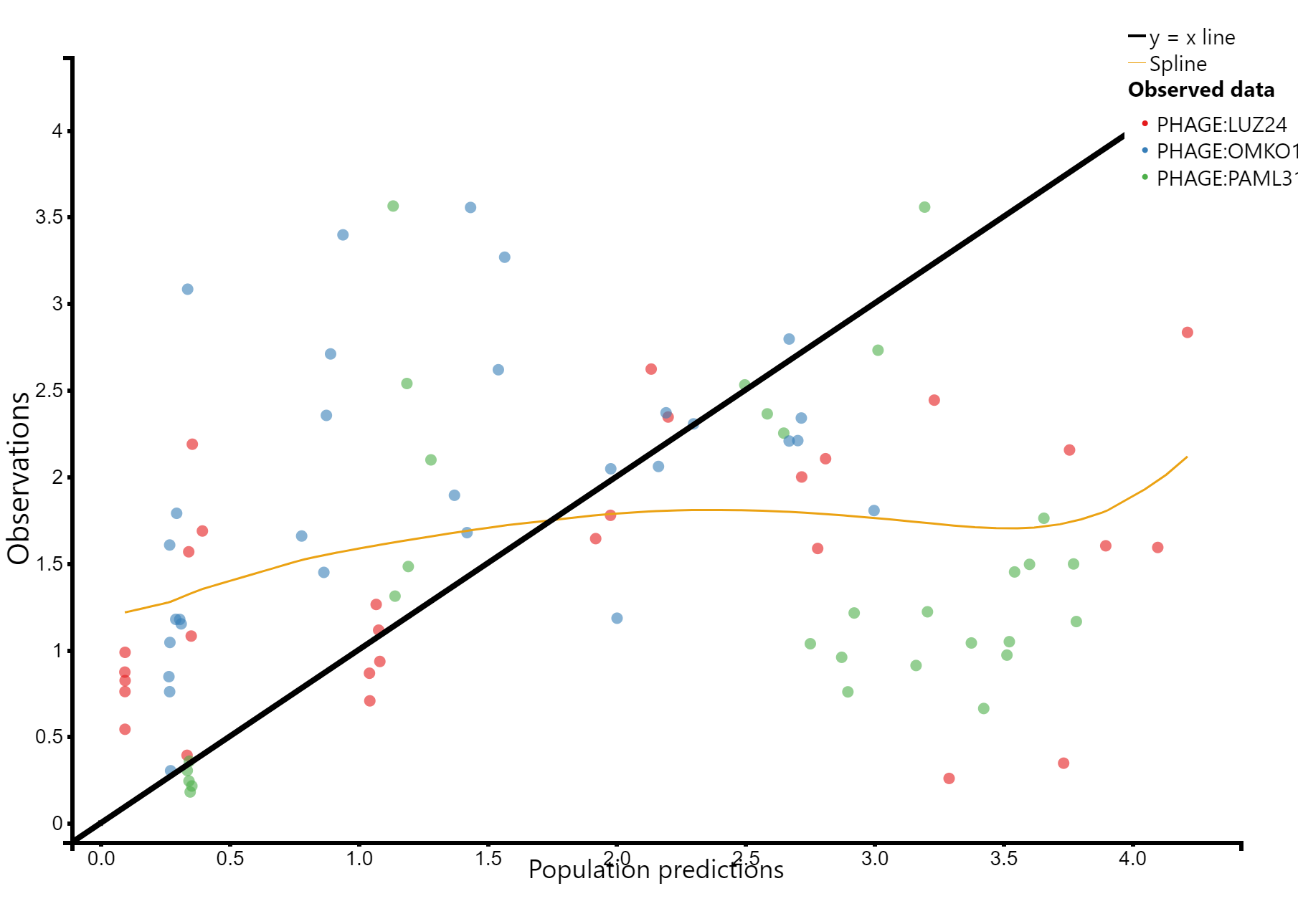


Figure S5: Observed versus predicted diagnostic plot of Liver measurements.


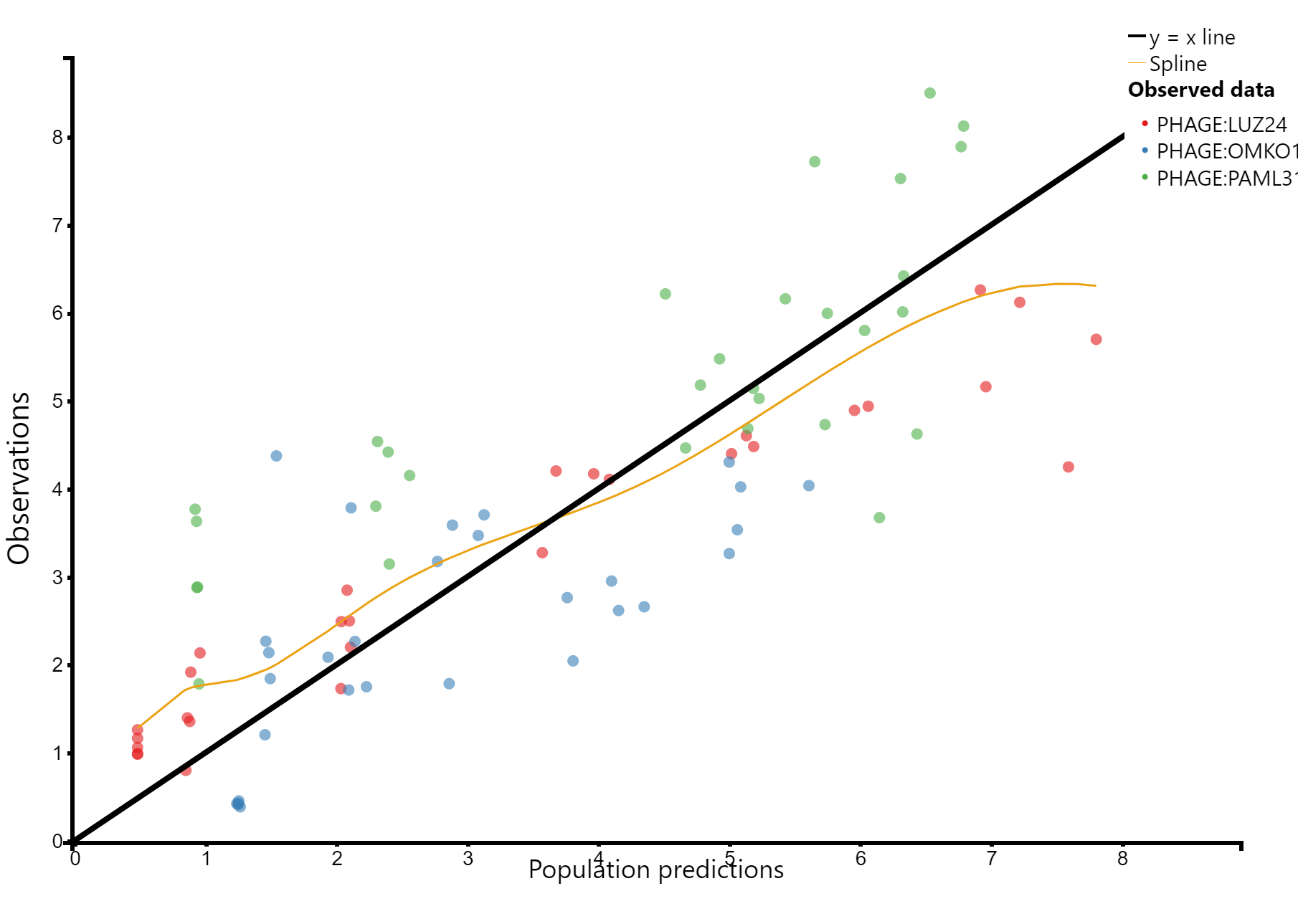


Figure S6: Observed versus predicted diagnostic plot of Lung measurements.


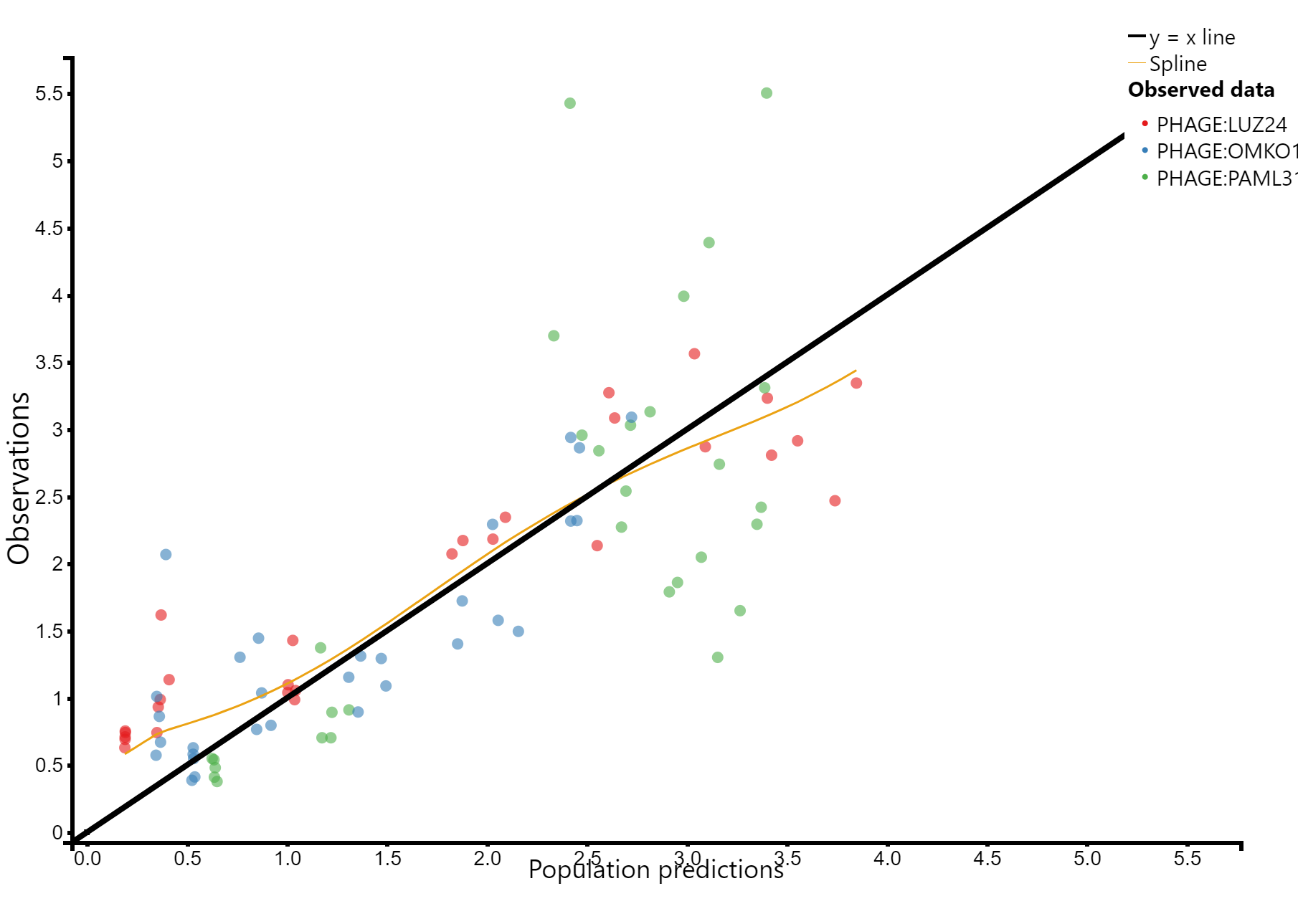


Figure S7: Observed versus predicted diagnostic plot of Muscle measurements.


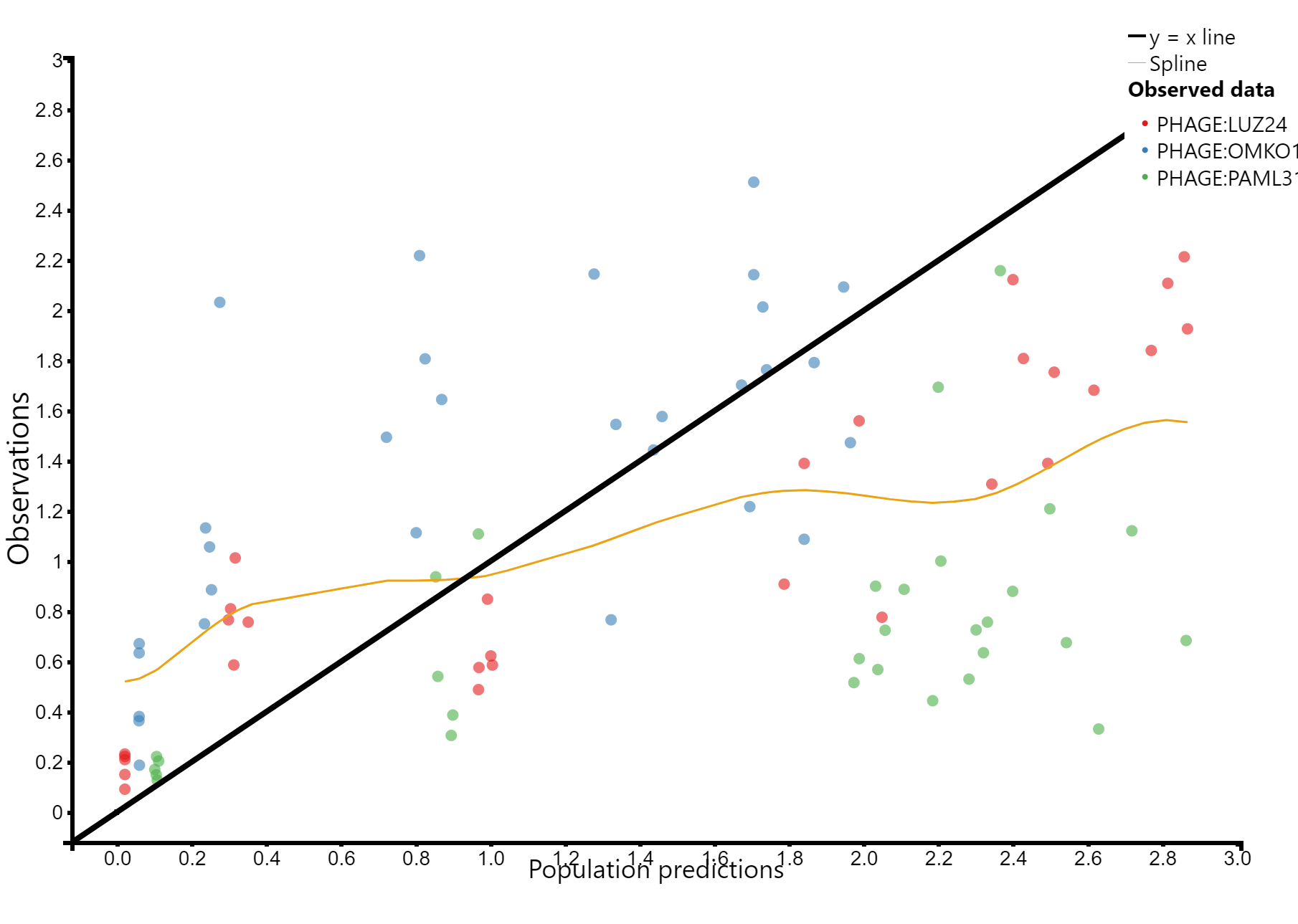


Figure S8: Observed versus predicted diagnostic plot of Sm. Intestines measurements.


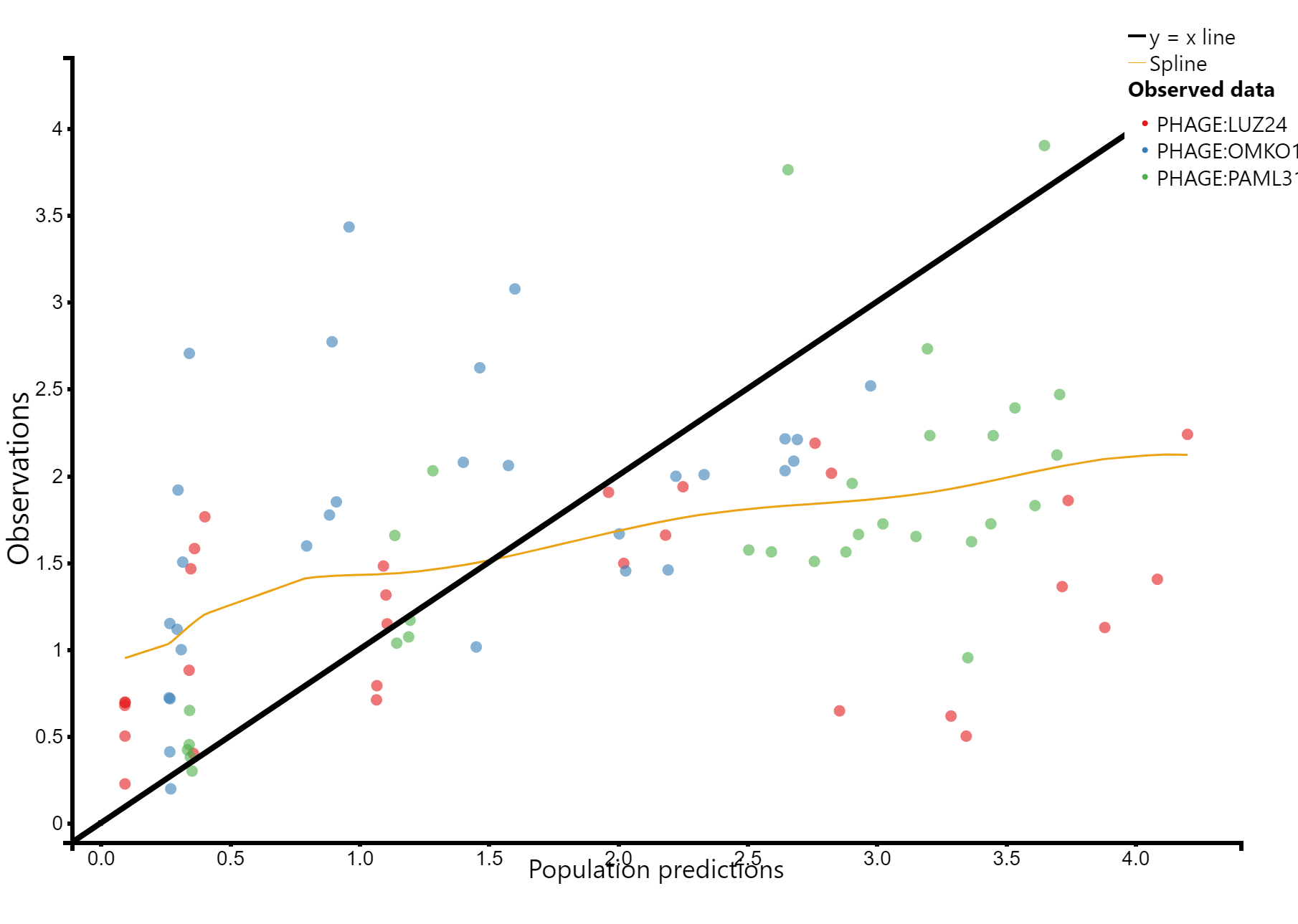


Figure S9: Observed versus predicted diagnostic plot of Spleen measurements.


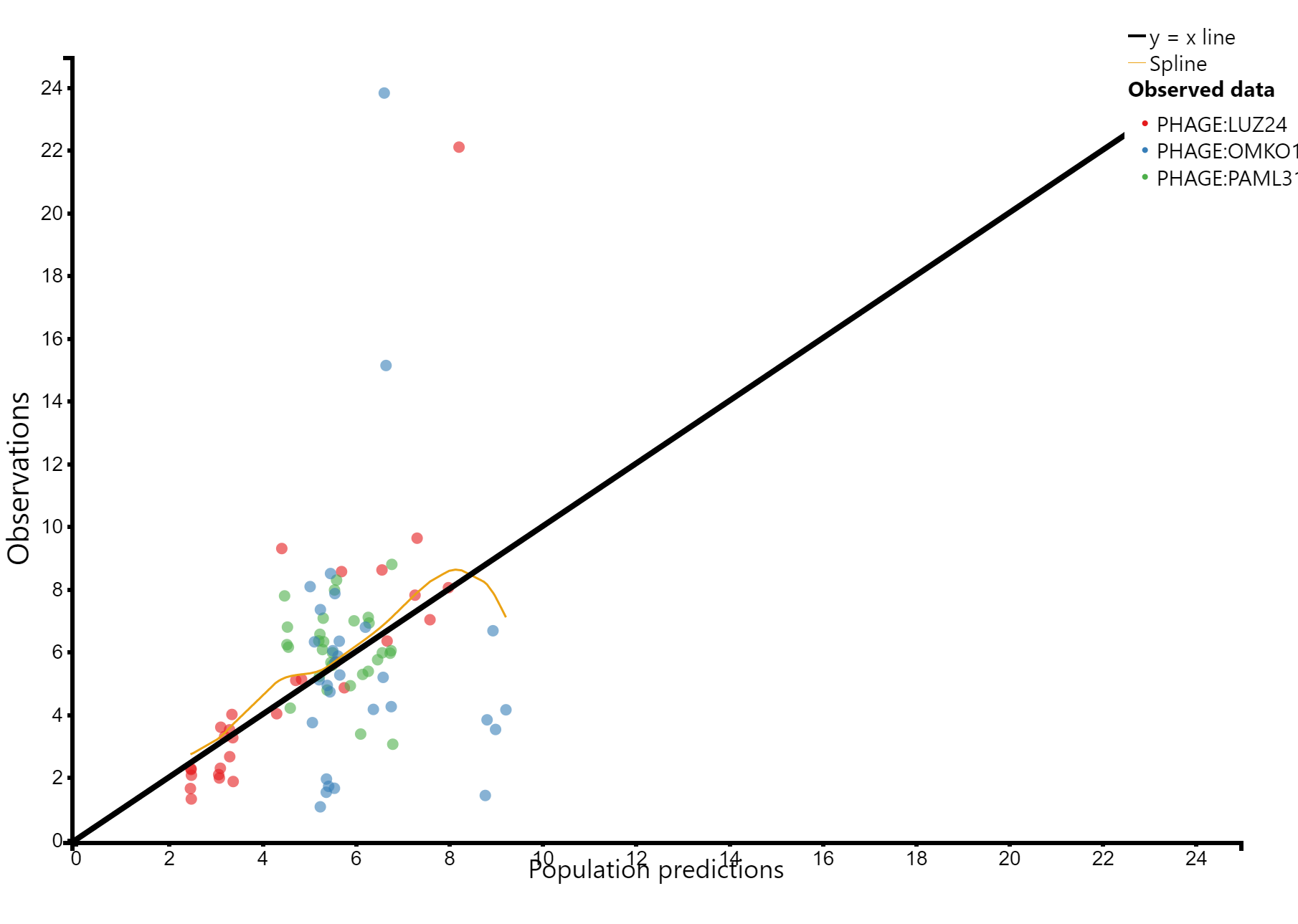


Figure S10: Observed versus predicted diagnostic plot of Stomach measurements.


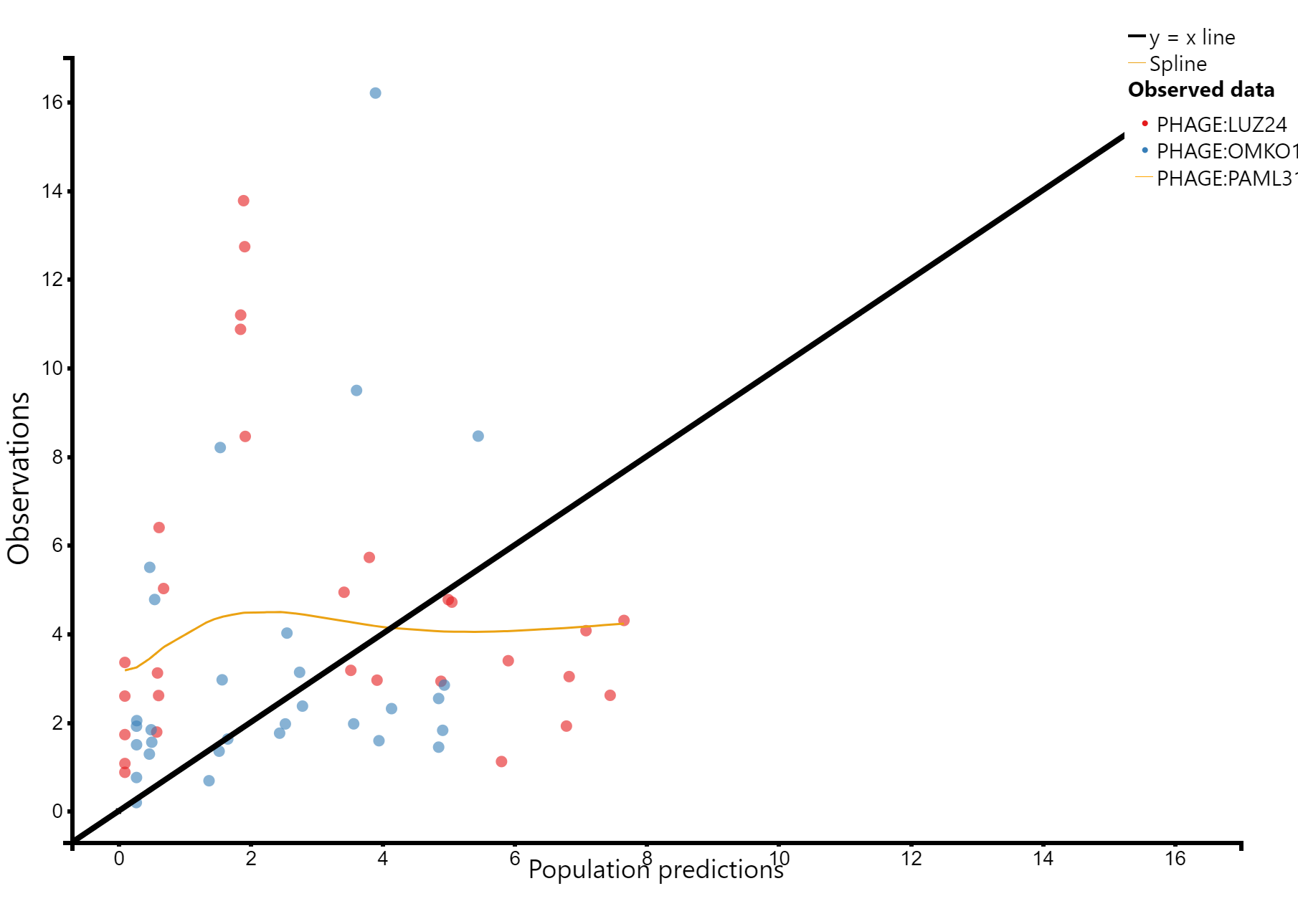


Figure S11: Observed versus predicted diagnostic plot of Stomach Contents measurements.


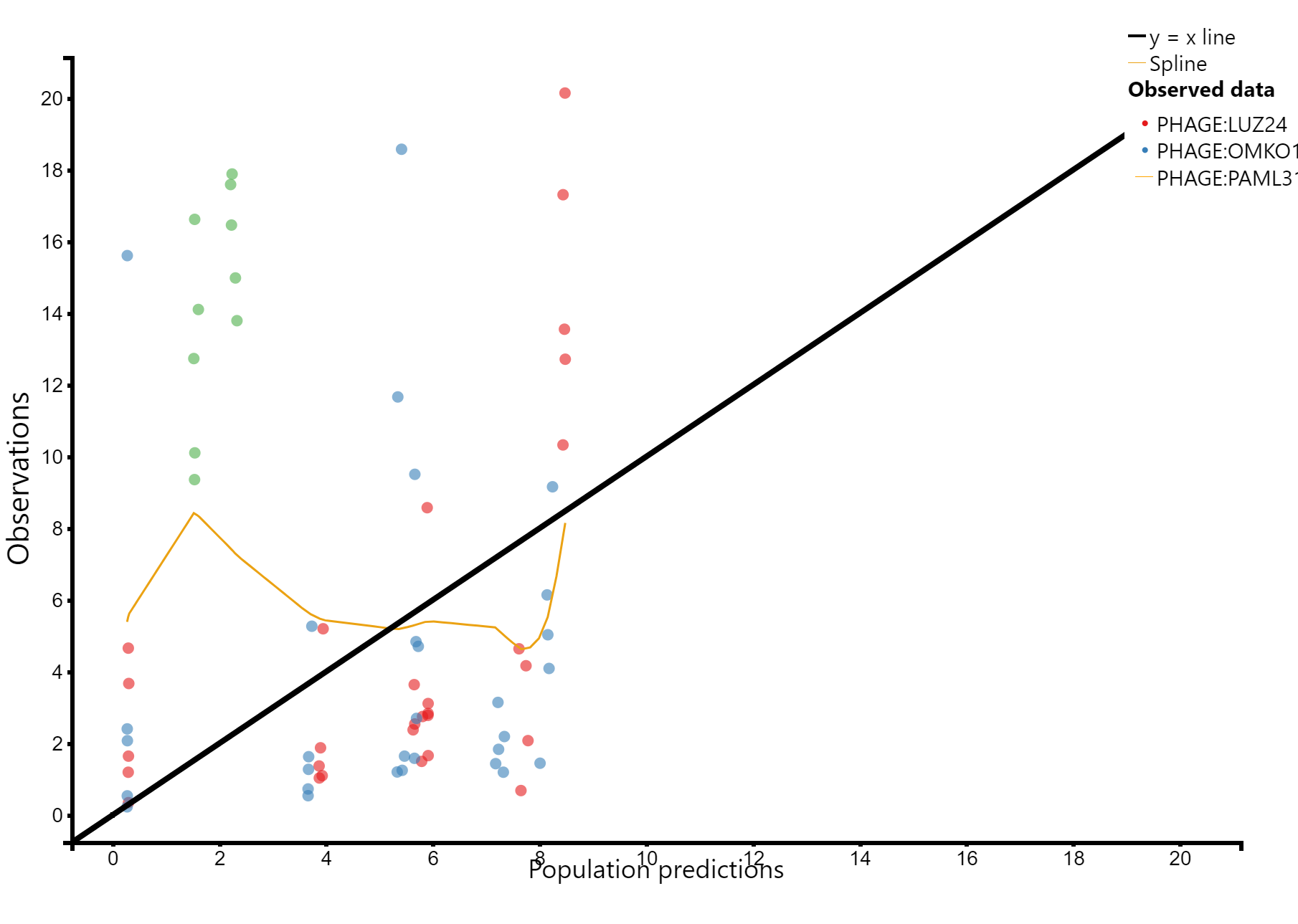


Figure S12: Observed versus predicted diagnostic plot of Urine measurements.


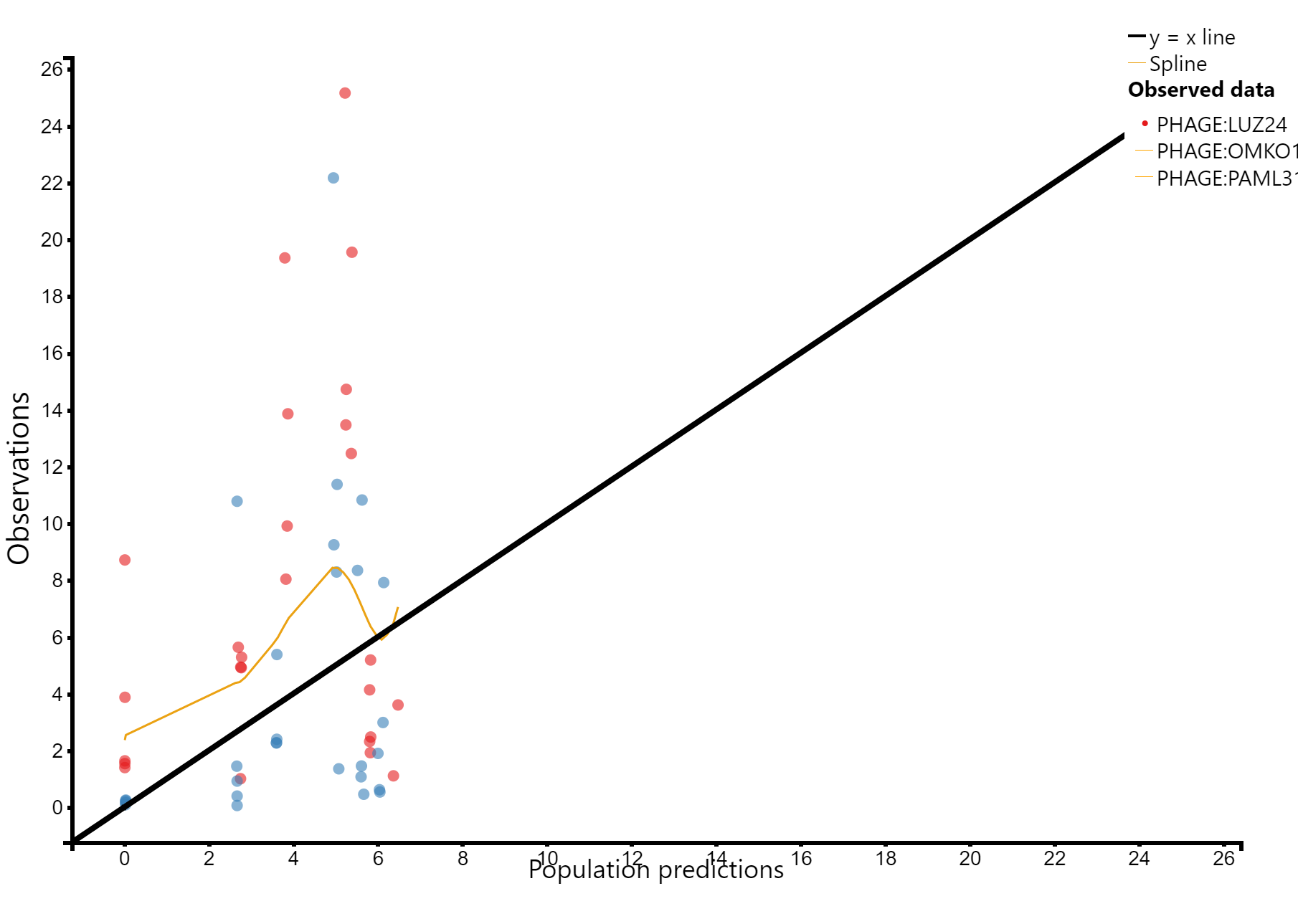


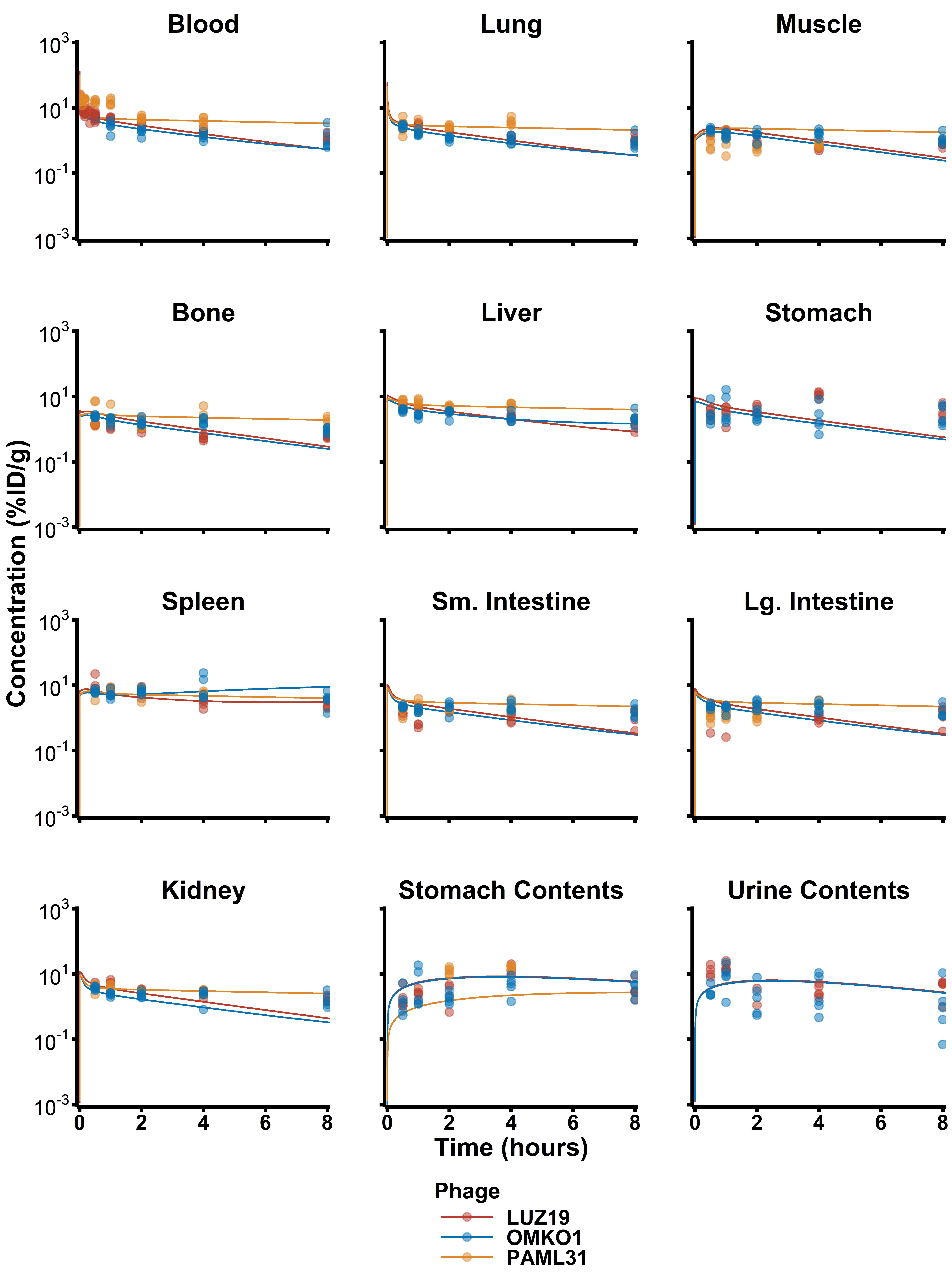
**Figure S13: PBPK Post Hoc fits, first 8 hours**

**Figure S14: Global Sensitivity Analysis (GSA) of key model parameters**


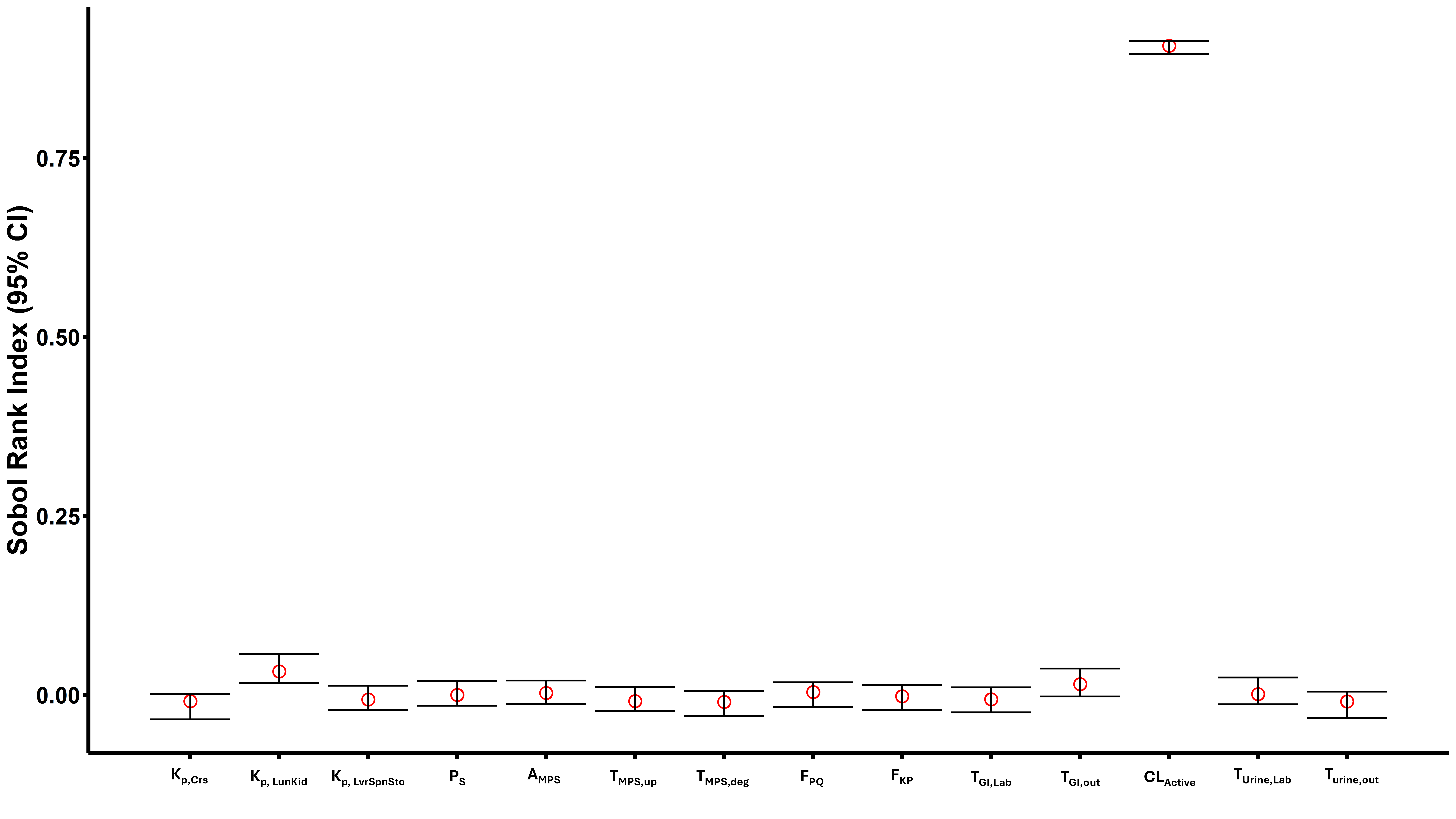


**Table S4: Global Sensitivity Analysis (GSA) of Key Model Parameters**

First order rank Sobol indices for each parameter based on 1000 simulated parameter sets and 95% confidence intervals.

|  | First-order rank | bias | std. error | min. c.i. | max. c.i. |
| --- | --- | --- | --- | --- | --- |
| K_p, Crs_ | -0.00849 | 0.00615 | 0.008808 | -0.03384 | 0.001178 |
| K_p, LunKid_ | 0.03295 | -0.00656 | 0.008808 | 0.016941 | 0.057171 |
| K_p, LvrSpnSto_ | -0.00609 | -0.00268 | 0.008281 | -0.02106 | 0.013013 |
| P_S_ | 0.000263 | -0.00248 | 0.007997 | -0.01481 | 0.019414 |
| A_MPS_ | 0.003032 | -0.00129 | 0.008496 | -0.01238 | 0.020325 |
| T_MPS, up_ | -0.00844 | -0.00105 | 0.007913 | -0.02215 | 0.011549 |
| T_MPS, deg_ | -0.00953 | 0.001508 | 0.00931 | -0.02941 | 0.006002 |
| F_PQ_ | 0.004273 | 0.003336 | 0.009516 | -0.01653 | 0.017865 |
| F_KP_ | -0.00182 | -0.0014 | 0.008408 | -0.02107 | 0.014114 |
| T_GI, Lab_ | -0.00596 | 0.001071 | 0.009045 | -0.02411 | 0.010836 |
| T_GI, out_ | 0.01513 | -0.00372 | 0.00977 | -0.00192 | 0.036951 |
| CL_Active_ | 0.907024 | 0.002831 | 0.004584 | 0.895669 | 0.913967 |
| T_Urine, Lab_ | 0.001327 | -0.00197 | 0.008923 | -0.01294 | 0.024457 |
| T_urine, out_ | -0.00898 | 0.00312 | 0.008724 | -0.03208 | 0.004765 |

**Figure S15. Predictions of Blood Phage concentrations in Kim et al (Lancet Infectious Diseases 2024)
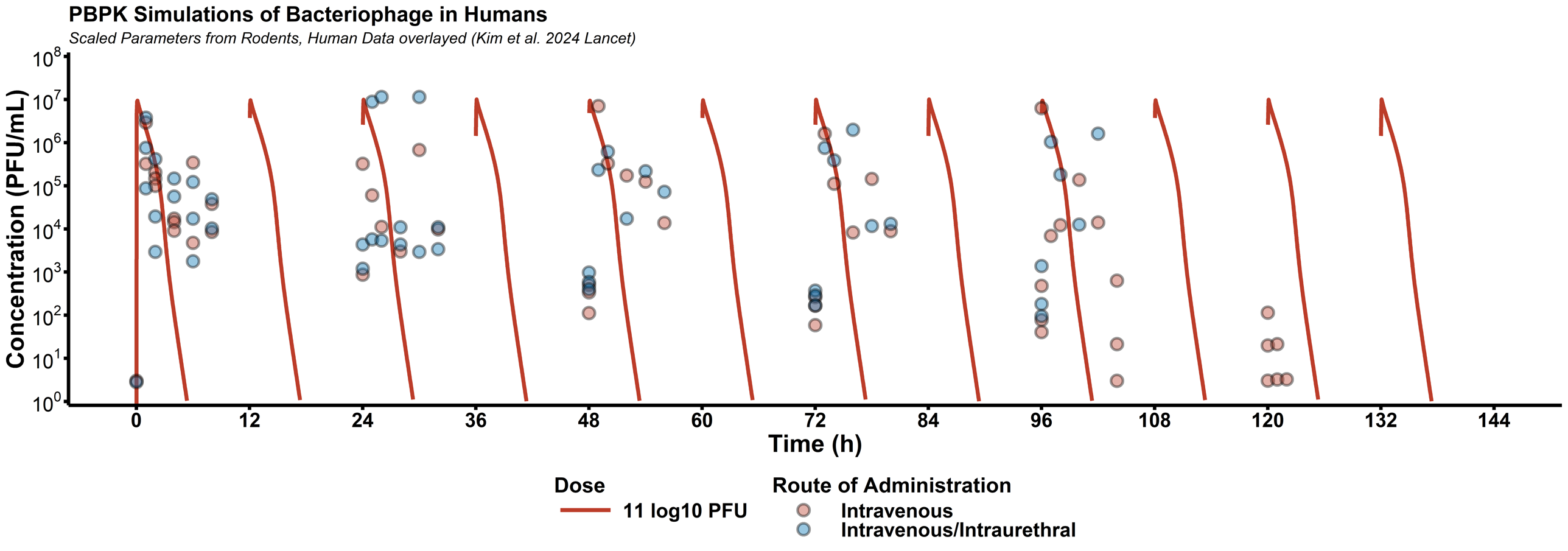
**

(1) Brown, R.P., Delp, M.D., Lindstedt, S.L., Rhomberg, L.R. & Beliles, R.P. Physiological parameter values for physiologically based pharmacokinetic models. *Toxicol Ind Health* **13**, 407-84 (1997).

(2) Buur, J.L., Baynes, R.E., Craigmill, A.L. & Riviere, J.E. Development of a physiologic-based pharmacokinetic model for estimating sulfamethazine concentrations in swine and application to prediction of violative residues in edible tissues. *Am J Vet Res* **66**, 1686-93 (2005).

(3) Buur, J., Baynes, R., Smith, G. & Riviere, J. Use of probabilistic modeling within a physiologically based pharmacokinetic model to predict sulfamethazine residue withdrawal times in edible tissues in swine. *Antimicrobial agents and chemotherapy* **50**, 2344-51 (2006).

(4) Crowell, S.R. *et al.* Preliminary physiologically based pharmacokinetic models for benzo[a]pyrene and dibenzo[def,p]chrysene in rodents. *Toxicol Appl Pharmacol* **257**, 365-76 (2011).

(5) Davies, B. & Morris, T. Physiological parameters in laboratory animals and humans. *Pharm Res* **10**, 1093-5 (1993).

(6) Carlander, U., Li, D., Jolliet, O., Emond, C. & Johanson, G. Toward a general physiologically-based pharmacokinetic model for intravenously injected nanoparticles. *Int J Nanomedicine* **11**, 625-40 (2016).

(7) Deng, L., Liu, H., Ma, Y., Miao, Y., Fu, X. & Deng, Q. Endocytosis mechanism in physiologically-based pharmacokinetic modeling of nanoparticles. *Toxicol Appl Pharmacol* **384**, 114765 (2019).

(8) Malik, P.R.V., Hamadeh, A. & Edginton, A.N. Model-Based Assessment of the Contribution of Monocytes and Macrophages to the Pharmacokinetics of Monoclonal Antibodies. *Pharm Res* **39**, 239-50 (2022).

(9) Sender, R. *et al.* The total mass, number, and distribution of immune cells in the human body. *Proc Natl Acad Sci U S A* **120**, e2308511120 (2023).

(10) Chou, W.-C., Cheng, Y.-H., Riviere, J.E., Monteiro-Riviere, N.A., Kreyling, W.G. & Lin, Z. Development of a multi-route physiologically based pharmacokinetic (PBPK) model for nanomaterials: a comparison between a traditional versus a new route-specific approach using gold nanoparticles in rats. *Particle and Fibre Toxicology* **19**, 47 (2022).
